# Decoding Hydrogen-Bond Network of Electrolyte for Cryogenic Durable Aqueous Zinc-Ion Batteries

**DOI:** 10.1007/s40820-025-01970-3

**Published:** 2026-01-03

**Authors:** Xiyan Wei, Jinpeng Guan, Yongbiao Mu, Yuhan Zou, Xianbin Wei, Lin Yang, Quanyan Man, Chao Yang, Limin Zang, Jingyu Sun, Lin Zeng

**Affiliations:** 1 Shenzhen Key Laboratory of Advanced Energy Storage, Department of Mechanical and Energy Engineering, Southern University of Science and Technology, Shenzhen, 518055 People’s Republic of China; 2 SUSTech Energy Institute for Carbon Neutrality, Southern University of Science and Technology, Shenzhen, 518055 People’s Republic of China; 3 College of Energy, Soochow Institute for Energy and Materials Innovations, Key Laboratory of Advanced Carbon Materials and Wearable Energy Technologies of Jiangsu Province, Soochow University, Suzhou, 215006 People’s Republic of China; 4 MOE Key Laboratory of New Processing Technology for Nonferrous Metal and Materials, Key Laboratory of Natural and Biomedical Polymer Materials (Education Department of Guangxi Zhuang Autonomous Region), College of Materials Science and Engineering, Guilin University of Technology, Guilin, 541004 People’s Republic of China; 5 Department of Materials Science and Engineering, Southern University of Science and Technology, Shenzhen, 518055 People’s Republic of China

**Keywords:** Aqueous zinc-ion batteries, Electrolyte additive, Hydrogen-bond reconstruction, High-rate performance, Low temperature

## Abstract

Aqueous zinc-ion batteries (AZIBs) hold great promise for next-generation energy storage but face challenges such as Zn dendrite growth, side reactions, and limited performance at low temperatures. Here, we propose an electrolyte design strategy that reconstructs the hydrogen-bond network through the synergistic effect of glycerol (GL) and methylsulfonamide (MSA), enabling the formation of a (100)-oriented Zn anode. This design significantly broadens the operating current and temperature windows of AZIBs. As a result, Zn||Zn symmetric cells exhibit remarkable cycling stability, achieving 4,000 h at 1 mA cm^−2^ and 600 h at 40 mA cm^−2^ (both at 1 mAh cm^−2^ capacity); even at −20 °C, Zn||Zn symmetric cells deliver ultra-stable cycling for over 5,400 h. Furthermore, Zn||VO_2_ full cells retain 77.3% of their capacity after 2,000 cycles at 30 °C with a current density of 0.5 A g^−1^ and 85.4% capacity retention after 2,000 cycles at −20 °C and 0.25 A g^−1^. These results demonstrate a robust pathway for enhancing the practicality and low-temperature adaptability of AZIBs.

**Supplementary Information:**

The online version contains supplementary material available at 10.1007/s40820-025-01970-3.

## Highlights

- The hydrogen-bond network structure and solvation structure of the electrolyte are reconstructed by glycerol (GL) and methylsulfonamide (MSA) to achieve low-temperature durability in aqueous zinc-ion batteries (AZIBs).
- GL and MSA collaboratively construct (100)-oriented high-activity dendrite-free zinc anode to improve the rate performance of AZIBs.
- The Zn||Zn symmetrical cell achieved stable operation for 4,000 h at 1 mA cm^−2^ and 1 mAh cm^−2^ (30 °C) and 5,400 h at 0.5 mA cm^−2^ and 0.5 mAh cm^−2^ (−20 °C).

**Supplementary Information:**

The online version contains supplementary material available at 10.1007/s40820-025-01970-3.

## Graphical abstract

**Figure:**
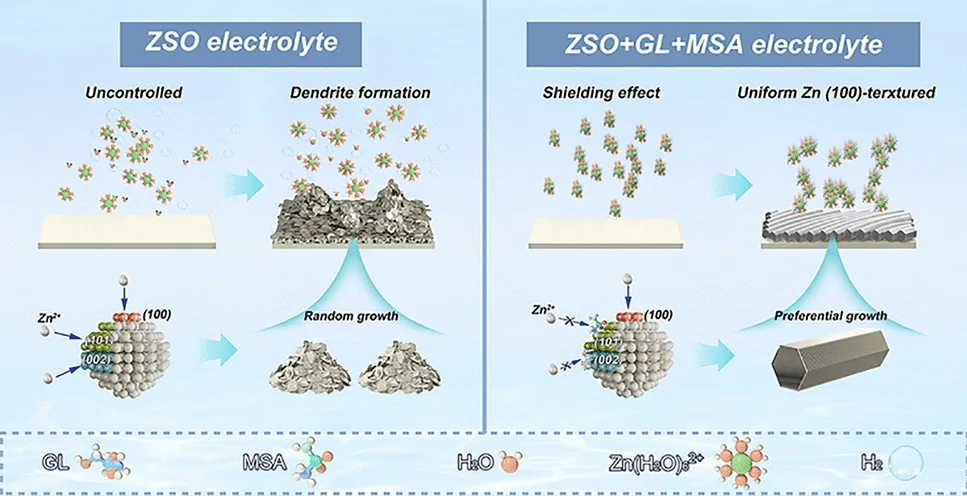

## Introduction

Aqueous zinc-ion batteries (AZIBs) have attracted considerable attention as promising candidates for large-scale grid energy storage systems owing to their intrinsic safety, high theoretical capacity [1], environmental friendliness, and low cost [2]. The zinc (Zn) anode, as the key component, exhibits excellent compatibility with aqueous electrolytes and offers a high theoretical capacity of 820 mAh g^−1^ [3]. However, Zn anodes suffer from disordered Zn deposition under uneven electric fields, leading to the rapid formation of dendrites [4–6]. Furthermore, the direct contact between water molecules and Zn anode induces severe parasitic side reactions, including hydrogen evolution reaction (HER) and corrosion, which degrade the interfacial stability and shorten the battery lifespan [7–9]. In addition, conventional aqueous electrolytes are susceptible to freezing at sub-zero temperatures, which severely restricts the operational temperature window of AZIBs [10, 11]. The sluggish interfacial kinetics of Zn also restricts its ability to support high-rate deposition and stripping, thereby narrowing the operable current range [12]. To address these challenges, it is crucial to develop novel electrolytes that simultaneously inhibit Zn dendrite formation, suppress electrolyte freezing, and enable high-rate operation [13–15]. Among various strategies, including anode surface engineering [16–19], separator modification [20–23], and electrolyte design [24–28], electrolyte optimization stands out as the most versatile approach. This is due to its tunability, which allows for simultaneous expansion of voltage [29–31], current [12, 32–34], and temperature windows [25, 35–39].

One effective route toward electrolyte optimization involves introducing functional additives [40]. However, single-component additives typically cannot balance stability with properties including conductivity, freezing point, and rate performance [41–43]. For example, many organic additives impair Zn ion transport due to inadequate shielding effects in single-solvent electrolytes, thereby improving battery life at the expense of ion mobility [44–46]. In contrast, multi-component additives are introduced, to not only integrate the advantages of individual additives but also enhance electrolyte entropy (∆S) [47–49]. According to thermodynamic principles (∆G = ∆H − T∆S), increasing ∆S can offset ∆H (enthalpic) penalties and lower the Gibbs free energy, thereby enhancing electrolyte stability and electrochemical reversibility [49–51]. This entropy enhancement is primarily associated with solvation structure reconfiguration, involving changes in the type, distribution, and proportion of coordinated solvent molecules and ions.

Furthermore, multi-component electrolytes elevate configurational entropy (*S*_*conf*_), increasing local structural disorder [41]. This enhanced disorder accelerates ion mobility and promotes a more homogeneous electric field and Zn^2+^ distribution (according to Eq. S4), thereby mitigating interfacial polarization and improving cycling stability [41]. In addition to improving stability, higher entropy suppresses the phase transition from disordered liquid to ordered solid, effectively lowering the freezing point of the electrolyte [52, 53]. This is because the freezing process of the electrolyte is the transition from an unordered liquid phase to an ordered solid phase. Maintaining the disorder of the electrolyte can inhibit its freezing, and the most direct way to increase the disorder of the electrolyte is by introducing multi-component additives to enhance the electrolyte’s ∆S [54]. Therefore, introducing multi-component additives increases the entropy of the electrolyte, not only enhancing stability of electrolyte but also lowering the freezing point of electrolyte by increasing its degree of disorder [55]. Additionally, introducing hydrogen-bond disruptors that interact with H_2_O, such as polyols, can construct the hydrogen-bond network with water and suppress free water activity, thereby improving low-temperature performance [56, 57].

Another critical factor for low-temperature performance is the construction of a highly active Zn anode, and highly active Zn anode accelerates the reaction kinetics of the electrode, which is not only conducive to easing the slow reaction kinetics at low temperature, but also improves the rate performance at room temperature. The electrochemical behavior of Zn is strongly influenced by its surface atomic configuration. In its hexagonal close-packed (*hcp*) structure, Zn exhibits different characteristics across crystallographic planes [58, 59]. The (002) plane has low surface energy and high dissolution activation energy due to its dense atomic arrangement, making it chemically stable but kinetically sluggish [60, 61]. In contrast, the (100) and (101) planes possess higher surface energy and lower activation barriers for Zn^2+^ stripping (1.16 and 1.24 eV vs. 1.72 eV for the (002) plane), rendering them more favorable for fast kinetics [62]. According to the Gibbs–Curie–Wulff theorem, crystal growth rates correlate with surface energy, thus favoring Zn growth along the (100) plane [63]. However, vertical growth of the (100) plane can exacerbate dendrite formation due to the “tip effect”, to ensure the stability of AZIBs, inducing leveling agent to guide the uniform deposition of Zn is necessary [64]. Therefore, the construction of a highly active and dendrite-free Zn anode necessitates the synergistic regulation through the use of multiple additives. Single additives often exhibit limited selectivity and control over Zn crystallization due to the intricate nature of Zn’s multiple crystal orientations. In contrast, multi-component additives promote cooperative adsorption on various Zn crystal facets, thereby facilitating preferential growth and effectively suppressing dendritic formation. This approach enhances the overall structural integrity and electrochemical performance of the Zn anode.

**Fig. 1:**
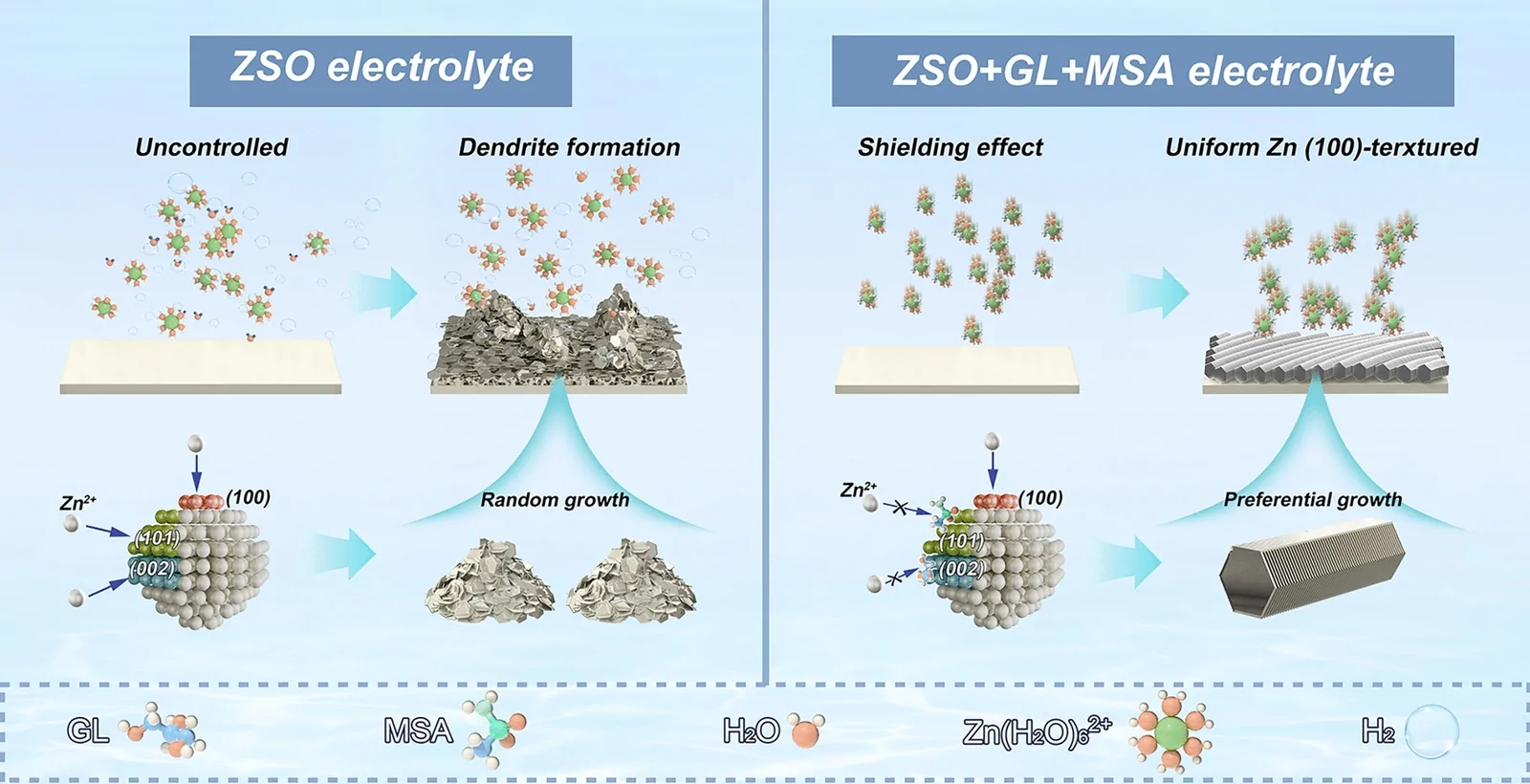
Schematic diagram of the mechanism of ZSO electrolyte and ZW_5_G_5_M_1_ electrolytes on Zn deposition behavior

In this study, as schematically illustrated in Fig. 1, we propose a dual-additive strategy by introducing glycerol (GL) and methylsulfonamide (MSA) into a 2 M ZnSO_4_ electrolyte. GL and MSA preferentially adsorb on the Zn (002) and Zn (101) planes, respectively, thereby suppressing Zn growth along these planes and promoting vertical growth along the highly active Zn (100) plane. This guided deposition leads to the formation of vertically aligned, flake-like Zn with a high degree of crystallographic orientation. Simultaneously, GL and MSA collaboratively reconstruct the hydrogen-bond network and solvation structure, increasing the configurational entropy of the electrolyte. This enhanced entropy facilitates homogeneous Zn^2+^ and electric field distribution, mitigating concentration polarization and compensating for the higher viscosity of GL. Additionally, the entropy-induced suppression of the liquid–solid transition improves low-temperature operation by inhibiting electrolyte freezing. On the Zn surface, GL forms an interfacial protective film, while MSA participates in the formation of a stable solid electrolyte interphase (SEI), effectively reducing H_2_O-induced side reactions and improving Zn reversibility and interfacial stability. As a result, Zn||Zn symmetric cells exhibit excellent electrochemical performance across a wide range of current densities and temperatures: 4,000 h stable cycling at 1 mA cm^−2^, 600 h at 40 mA cm^−2^ (both at 1 mAh cm^−2^ capacity), and a stable cycle of 5,000 h at 0.25 mA cm^−2^ and 0.25 mAh cm^−2^ even at the extreme -20 °C. Furthermore, Zn||VO_2_ full cells retain 77.3% capacity after 2,000 cycles at 30 °C (0.5 A g^−1^) and 85.4% at −20 °C (0.25 A g^−1^), highlighting the broad applicability and robustness of this electrolyte design. These findings offer a comprehensive and effective strategy for expanding the temperature and current operation windows of AZIBs, paving the way for their practical deployment in diverse energy storage scenarios.

## Experimental Section

### Materials

Zinc (Zn) foils (99.99%, 10 and 100 µm thickness) and titanium (Ti) foils (99.99%, 10 µm thickness) were purchased from Shenzhen Kejing Star Technology Co., Ltd. N-methyl-2-pyrrolidone (NMP), vanadium dioxide (VO_2_), and zinc sulfate heptahydrate (ZnSO_4_·7H_2_O) were obtained from Shanghai Macklin Biochemical Co., Ltd. Super P conductive carbon black was supplied by Jiangsu Shenzhou Carbon Co., Ltd. Methylsulfonamide (MSA) and glycerol (GL) were procured from Shanghai Aladdin Biochemical Technology Co., Ltd. All chemicals and materials used were of analytical grade and were utilized without any further purification.

### Preparation of Electrolyte

The 2 M ZnSO_4_ (ZSO) electrolyte was prepared by dissolving ZnSO_4_·7H_2_O in deionized water using a 100-mL volumetric flask. The ZW_a_G_b_M_c_ electrolytes were formulated by mixing ZnSO_4_·7H_2_O, deionized water, GL, and MSA. Initially, GL and deionized water were combined in volume ratios of 1:9, 2:8, 3:7, 4:6, and 5:5. Subsequently, ZnSO_4_·7H_2_O and MSA at various concentrations were added to the mixture, followed by continuous stirring until a homogeneous electrolyte solution was obtained.

### Characterizations

The morphologies of the samples were examined using a Hitachi SU-8230 field-emission scanning electron microscope (FE-SEM). Transmission electron microscopy (TEM), energy-dispersive X-ray spectroscopy (EDX), and elemental mapping were carried out on a Thermo Fisher Talos microscope operated at an acceleration voltage of 300 kV. X-ray photoelectron spectroscopy (XPS) was conducted using a Thermo Scientific ESCALAB 250Xi system with an Al Kα radiation source (hν = 1,486.8 eV). Raman spectra were collected using a HORIBA LabRAM HR Evolution spectrometer with a 532 nm laser as the excitation source. X-ray diffraction (XRD) patterns were recorded on a Bruker D8 Advance diffractometer equipped with a D/Tex Ultra detector and Cu-Kα radiation, operated at a scan rate of 5° min^−1^, to analyze the crystalline structure of the samples. Specifically, the in situ observation cells were assembled using molds purchased from Beijing Scistar Technology Co., Ltd., with both the working and counter electrodes composed of Zn metal. The electrolytes used were ZSO and ZW_5_G_5_M_1_. The Zn electrodes had an area of 0.1 cm^2^ and a thickness of 100 μm. An electrochemical workstation was employed to supply the power, and a constant current density of 10 mA cm^−2^ was applied during the experiments. These additional details ensure the reproducibility and transparency of the experimental procedure.

### Electrochemical Performance Assessment

Zn||Zn symmetric cells were assembled by sandwiching a glass fiber separator (Whatman GF/D) between commercial Zn plates (10 mm in diameter) in CR2025-type coin cells, which were filled with 70 μL of the respective electrolytes. Zn||Ti half-cells were assembled using Zn plates (10 mm in diameter) as the anode, Ti foils (16 mm in diameter) as the cathode, and GF/D glass fiber as separators. These cells were also assembled in CR2025-type coin cells and filled with 70 μL of different electrolytes. Zn||VO_2_ full cells were fabricated using Zn plates (14 mm in diameter) as the anode and VO_2_ electrodes (12 mm in diameter) as the cathode, with 80 μL of either ZSO or ZW_5_G_5_M_1_ electrolytes. The VO_2_ cathode was prepared by mixing VO_2_, Super P carbon black, and PVDF binder in a mass ratio of 8:1:1, using NMP as the solvent. The slurry was stirred for 12 h, cast onto Ti foil, and dried at 70 °C. The VO_2_ loading was ranged from 0.5 to 1.5 mg per electrode. For pouch cell assembly, Zn plates (40 mm × 30 mm) were used as the anode, and VO_2_ electrodes (25 mm × 35 mm) were used as the cathode. The pouch cells were filled with 300 μL of ZW_5_G_5_M_1_ electrolyte. Nickel strips were attached to both electrodes using conductive tape. The cell stack, consisting of cathode, separator, and anode, was enclosed in an aluminum–plastic film, which was heat-sealed to ensure proper encapsulation. An additional 300 μL of electrolyte was injected into the sealed cell. To evaluate the electrochemical performance and coulombic efficiency (CE) of Zn plating/stripping, Zn||Ti asymmetric cells were tested using a Neware battery test system (Shenzhen, China) at 30 °C with ZSO and ZW_5_G_5_M_1_ electrolytes. For CE measurements, 1 mAh cm^−2^ of Zn was plated onto Ti foil and subsequently stripped to 0.6 V in each cycle under current densities of 10 mA cm^−2^. Cycling stability and voltage hysteresis were evaluated using Zn||Zn symmetric cells tested at various current densities (1–60 mA cm^−2^) with a fixed Zn deposition capacity of 0.25–5 mAh cm^−2^. Electrochemical impedance spectroscopy (EIS) was performed on a CHI760d electrochemical workstation (CH Instruments, Shanghai, China) over a frequency range of 100 kHz to 100 mHz. Chronoamperometry (CA) measurements were conducted under a fixed overpotential of 0.15 V. Cyclic voltammetry (CV) tests were performed on Zn||Ti cells in both ZSO and ZW_5_G_5_M_1_ electrolytes over a voltage range of 1 to −0.3 V at a scan rate of 1 mV s^−1^. Linear sweep voltammetry (LSV) was carried out using Zn||Zn symmetric cells in ZSO and ZW_5_G_5_M_1_ electrolytes at a scan rate of 1 mV s^−1^.

### DFT and MD Calculation Method

Classical molecular dynamics (MD) simulations were conducted to investigate the mixed electrolytes at the atomic scale. One bulk model (System1) was constructed for these simulations. System 1 comprises 200 Zn^2+^ ions, 200 SO_4_^2−^ ions, 100 methylsulfonamide (MSA) molecules, and 431 glycerol (GL) molecules. The initial configuration of the system was generated using the PACKMOL software, with all species randomly placed within a cubic simulation box. The partial charges of all molecules were calculated using the Gaussian 16 software package, employing the 6-311G (d, p) basis set. Due to the high concentration of zinc sulfate in the system, the charges of the Zn^2+^ and SO_4_^2−^ ions were scaled by a factor of 0.8 to account for overestimated electrostatic interactions. The OPLS-AA force field was applied to describe the interactions of ZnSO_4_ and the target organic molecules, while the TIP3P model was used for water molecules. The molecular force field includes both bonded and non-bonded interactions. The non-bonded interactions consist of van der Waals (vdW) forces and electrostatic interactions, which are represented by Eqs. (1) and (2), respectively.

1 $$\begin{array}{*{20}c} {E_{LJ} \left( {r_{ij} } \right) = 4\varepsilon_{ij} \left( {\left( {\frac{{{\upsigma }_{ij} }}{{r_{ij} }}} \right)^{12} - \left( {\frac{{{\upsigma }_{ij} }}{{r_{ij} }}} \right)^{6} } \right)} \\ \end{array}$$

2 $$\begin{array}{*{20}c} {E_{c} \left( {r_{ij} } \right) = \frac{{q_{i} q_{j} }}{{4\pi \varepsilon_{o} \varepsilon_{r} r_{ij} }}} \\ \end{array}$$

In the equation, $q_{i}$, $q_{j}$ are atomic charge, $r_{ij} { }$ is the distance between atoms, ${\upsigma }$ is the atomic diameter, and ε is the atomic energy parameter.

For different kinds of atoms, the Lorentz–Berthelot mix rules were adopted for vdW interactions, which follows Eq. (3). The cutoff distance of vdW and electronic interactions was set to 1.2 nm, and the PME method was employed to calculate long-range electrostatic interactions.

3 $$\begin{array}{*{20}c} {\sigma_{ij} = \frac{1}{{2\left( {\sigma_{ii} + \sigma_{jj} } \right)}};\varepsilon_{ij} = \left( {\varepsilon_{ii} {*}\varepsilon_{jj} } \right)^{\frac{1}{2}} } \\ \end{array}$$

For the MD simulations, energy minimization was first conducted to relax the initial structure of the simulation box. Subsequently, an isothermal-isobaric (NPT) ensemble was employed with a time step of 1.0 fs to further optimize the simulation box, maintaining the temperature at 298.15 K and the pressure at 1.0 atm. The duration of the NPT equilibration was set to 20.0 ns, which is sufficient to achieve a stable box size. Throughout all MD simulations, atomic motion was governed by classical Newtonian mechanics, and the equations of motion were integrated using the velocity-Verlet algorithm. All MD simulations were performed using the GROMACS 2021.5 software package.

Density functional theory (DFT) calculations for water, salt, and target molecules were carried out using the Gaussian software. Implicit solvation effects were accounted for using the solvation model based on density (SMD). Molecular structures were visualized with Visual Molecular Dynamics (VMD). The binding energy (*E*_Binding_) of the complexes was calculated according to Eq. (4):

4 $$\begin{array}{*{20}c} {E_{Binding} = E_{{{\mathrm{complexe}}1 - {\mathrm{complexe}}2}} - E_{{{\mathrm{complexe}}1}} - E_{{{\mathrm{complexe}}2}} } \\ \end{array}$$

where $E_{{{\mathrm{complexe}}1 - {\mathrm{complexe}}2}}$ represents the total energy of the ${\mathrm{complexe}}1$ interacting ${\mathrm{complexe}}2$. $E_{{{\mathrm{complexe}}1}}$ is the energy of the ${\mathrm{complexe}}1$, and $E_{{{\mathrm{complexe}}2}}$ is the energy of the ${\mathrm{complexe}}2$.

## Results and Discussion

### Effect of GL and MSA on the Solvation Structure and Hydrogen Bonding Network

In this study, the benchmark electrolyte composed of 2 M ZnSO_4_ is denoted as ZSO, while modified electrolytes with optimized compositions are referred to as ZW_a_G_b_M_c_, in which **Z** indicates the presence of 2 M ZnSO_4_, **a** represents ten times the volume ratio of water, **b** represents ten times the volume ratio of glycerol (GL), and **c** denotes the concentration of MSA in the electrolyte (in mol). The electrostatic potential maps of MSA and GL (Fig. S1) reveal that the regions near the S=O group in MSA and the hydroxyl oxygen in GL exhibit highly negative potential. These negatively charged regions are potential Zn^2+^-affinitive sites and also serve as active sites for hydrogen bonding with the hydrogen atoms in H_2_O molecules.

**Fig. 2:**
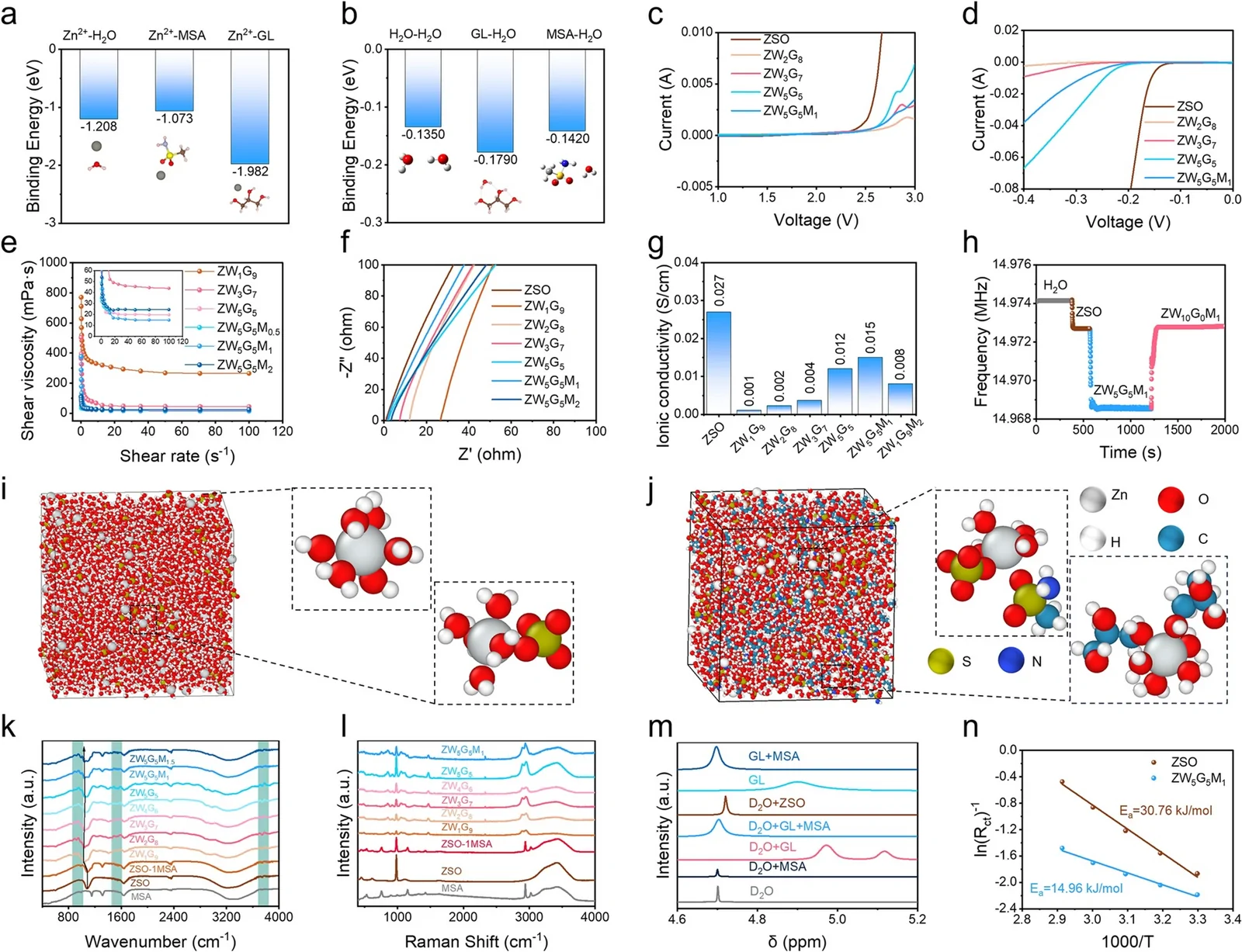
The binding energy of **a** Zn^2+^ and each component, **b** H_2_O and each component. **c** LSV curves, **d** HER tests of Zn||Zn symmetric cells assembled with different electrolytes. **e** Viscosity of different electrolytes. **f** Impedance of SS||SS cells assembled with different electrolytes. **g** Ionic conductivity corresponding to the impedance curves in (**f)**. **h** QCM tests with different liquids. MD simulations and solvation structure of **i** ZSO electrolyte, **j** ZW_5_G_5_M_1_ electrolyte. **k** FTIR spectra, **l** Raman spectra, **m** Nuclear magnetic hydrogen spectra of different liquids. **n** Activation energy of ZSO and ZW_5_G_5_M_1_ electrolytes

Binding energy calculations between Zn^2+^ and H_2_O, GL, and MSA (Fig. 2a) show that Zn^2+^ has a lower binding energy with GL than with H_2_O, indicating that Zn^2+^ preferentially coordinates with GL. This suggests that GL molecules partially replace H_2_O in the Zn^2+^ solvation shell, reducing the number of coordination water to inhibit the HER. Binding energy analysis between H_2_O and other components (Fig. 2b) shows that GL exhibits the strongest interaction with H_2_O, implying its capability to disrupt the H_2_O hydrogen-bond network and reconstruct it through strong GL-H_2_O interactions. This reconstruction increases electrolyte disorder and decreases free H_2_O activity, thus hindering the phase transition from a disordered liquid to an ordered solid and lowering the electrolyte’s freezing point.

The reduced coordination number of H_2_O in the solvation shell also suppresses water-induced parasitic reactions, such as the HER. However, the inherently high viscosity of GL leads to severe Zn^2+^ concentration gradients, which may aggravate dendrite growth. According to Eq. S4, an increase in *S*_*conf*_ enhances ion mobility, enabling faster response to electric field fluctuations and improving ion transport efficiency. Therefore, MSA is introduced to increase *S*_*conf*_ and strengthen H_2_O binding, further enhancing ion mobility and reducing HER. As demonstrated in Fig. 2c, d, increasing GL content and incorporating MSA extend the stable electrochemical window, confirming the effectiveness of GL and MSA in suppressing HER. However, GL significantly increases electrolyte viscosity. As shown in Fig. 2e, the viscosity of ZW_1_G_9_ (90 vol% GL) reaches approximately 300 mPa·s at a shear rate of 36 s^−1^. Reducing the GL content to 50 vol% (ZW_5_G_5_) lowers viscosity to ~ 20 mPa·s under the same conditions. When MSA is introduced into ZW_5_G_5_, the viscosity initially increases, then decreases, and increases again. This behavior results from the interplay between electrolyte concentration and configurational entropy. At low MSA content, the binding energy between Zn^2+^ and GL exceeds that of Zn^2+^ and MSA, and due to the abundance of GL and H_2_O, MSA cannot effectively enter the solvation structure, resulting in minimal entropy gain. As the MSA concentration increases, its ability to alter the solvation structure and hydrogen-bond network becomes significant, boosting *S*_*conf*_ and thereby reducing viscosity. Beyond optimal concentration, however, further MSA addition increases overall electrolyte concentration and viscosity once again. Changes in viscosity correlate with the ionic conductivity of the electrolyte. As shown in Fig. 2f, g, although GL reduces ionic conductivity due to increased viscosity, the introduction of an appropriate amount of MSA compensates for this effect by enhancing *S*_*conf*_ and ion mobility. Balancing these factors, the electrolyte composition ZW_5_G_5_M_1_ is identified as optimal, achieving an ideal trade-off between ionic conductivity and HER suppression. After confirming the appropriate electrolyte composition, the anti-freezing performance of the electrolyte were evaluated by differential scanning calorimetry (DSC). The DSC results shown in Fig. S2 indicate that the ZSO electrolyte exhibits a phase transition signal near 0 °C, while the ZW_5_G_5_M_1_ electrolyte displays its phase transition signal around −45 °C. Additionally, as shown in Fig. S3, we observe that the ZSO electrolyte solidifies and freezes at −20 °C, whereas the ZW_5_G_5_M_1_ electrolyte remains in a liquid state. Both the DSC results and low-temperature observe experiments demonstrate that GL and MSA effectively lower the freezing point of the electrolyte.

To further evaluate the electrolyte’s capacity to suppress HER, a quartz crystal microbalance (QCM) was employed, through which different liquids were passed at a constant flow rate of 100 μL min^−1^. By monitoring the vibration frequency of a Zn- based QCM chip, the mass of adsorbed species was determined. As shown in Fig. 2h, the substitution of water with the ZSO electrolyte led to a decrease in vibration frequency, attributable to the adsorption of electrolyte ions onto the Zn surface. When ZSO was replaced by the ZW_5_G_5_M_1_ electrolyte, a more pronounced frequency decrease was observed, indicating significantly greater adsorption of species onto the Zn surface in the presence of ZW_5_G_5_M_1_. Conversely, replacing ZW_5_G_5_M_1_ with the ZW_10_G_0_M_1_ electrolyte yielded a frequency change similar to that of ZSO, suggesting that the predominant adsorbed component in ZW_5_G_5_M_1_ is GL. This adsorption of GL on the Zn surface effectively reduces direct contact between water molecules and Zn, thereby mitigating HER. The preferential adsorption behavior was further confirmed by calculating the adsorption energies of MSA, GL, and H_2_O on the Zn surface (Fig. S4). Both GL and MSA exhibit lower adsorption energies than H_2_O, indicating a stronger tendency to bind to Zn and thus replace water molecules, ultimately reducing corrosion and HER on the Zn anode. Since MSA and GL modulate the Zn^2+^ solvation environment, the coordination number of H_2_O molecules within the Zn^2+^ solvation shell was further analyzed. MD simulations were conducted for both the ZSO (Fig. 2i) and ZW_5_G_5_M_1_ (Fig. 2j) electrolytes, with coordination numbers provided in Fig. S5. In the ZSO electrolyte, Zn^2+^ ions are predominantly surrounded by H_2_O molecules, while in the ZW_5_G_5_M_1_ electrolyte, a portion of the H_2_O molecules are replaced by GL and MSA, reducing the H_2_O coordination number from 5.73 to 4.32. Prior studies by Wang et al. have shown that HER is primarily driven by coordinated water molecules migrating with Zn^2+^ ions [65]. Therefore, decreasing the number of coordinated H_2_O molecules is an effective strategy for suppressing HER, further validating the synergistic role of GL and MSA in enhancing Zn anode stability. Therefore, reducing the number of coordinated H_2_O molecules can effectively suppress HER, the above calculation results and theoretical proof demonstrate that the introduction of MSA and GL can effectively suppress the occurrence of HER.

The influence of GL and MSA on the hydrogen-bond network was examined using Fourier-transform infrared (FTIR) spectroscopy (Fig. 2k). GL exhibits characteristic peaks at 827.4–956.7, 2,854.4–3,043.2, and 3,681.8–3,824.5 cm^−1^, while MSA shows distinct peaks in the 1,114.7–1,361.6 cm^−1^ region. The -SO_3_ stretching peak appears between 1,000 and 1,200 cm^−1^ and shifted to lower wavenumbers upon GL and MSA addition, indicating their incorporation into the solvation sheath and enhanced Zn^2+^-SO_4_^2−^ interactions. Moreover, the O–H stretching vibrations (2,800–3,600 cm^−1^) shifted to higher wavenumbers, suggesting that GL and MSA disrupt the hydrogen-bond network of water, thereby increasing electrolyte disorder. Raman spectroscopy further revealed structural changes in the hydrogen-bond network following GL and MSA incorporation [66]. As illustrated in Fig. 2l, MSA exhibits peaks at 751.4, 1,154.3, 2,939.4, and 3,027.3 cm^−1^, while GL showed characteristic peaks at 847.6, 1,469.7, and 2,846.1–3,010.2 cm^−1^. Notably, the 2,900–3,700 cm^−1^ region exhibits substantial shifts, affirming the reorganization of the hydrogen-bond network. Additionally, nuclear magnetic resonance (NMR) spectra (Fig. 2m) showed signal shifts in the presence of GL and MSA, providing further evidence of hydrogen-bond network reconstruction. The activation energy (*E*_a_) of the electrolyte is intrinsically related to its solvation structure. Based on temperature-dependent conductivity data (Fig. S6), *E*_a_ was calculated (Fig. 2n). The ZW_5_G_5_M_1_ electrolyte exhibits a significantly lower *E*_a_ (14.96 kJ mol^−1^) compared to ZSO (30.76 kJ mol^−1^), indicating a more favorable Zn^2+^ desolvation process, further confirming solvation structure modulation by GL and MSA.

### GL and MSA Construct the SEI

**Fig. 3:**
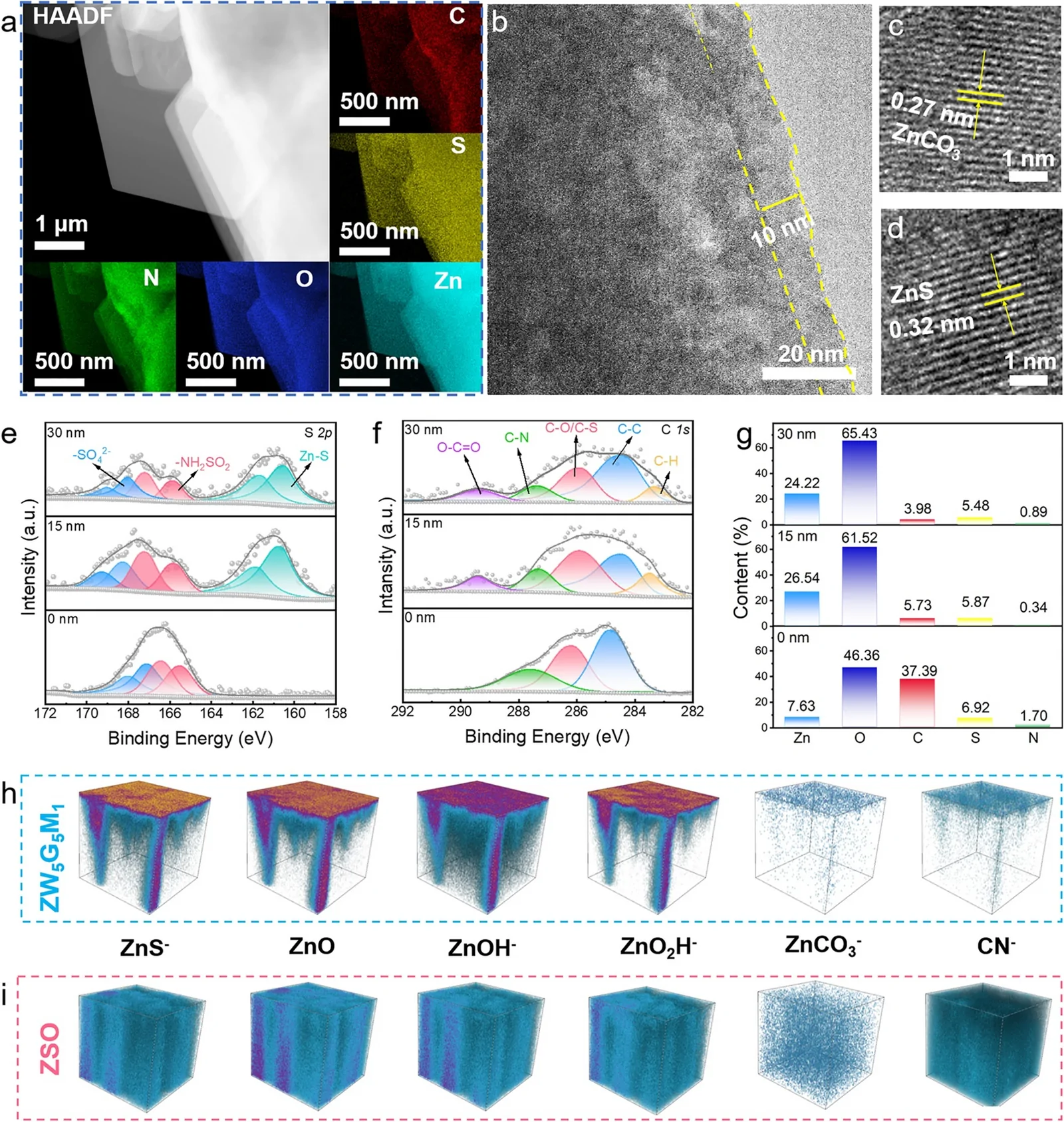
**a** Transmission electron microscopy (TEM mapping image of Zn deposited with ZW_5_G_5_M_1_ electrolyte. **b** High-resolution TEM image of Zn deposited with ZW_5_G_5_M_1_ electrolyte. TEM of **c** ZnCO_3_ and **d** ZnS crystal phase. XPS etching of Zn after cycling with ZW_5_G_5_M_1_ electrolyte, **e** S *2p*, **f** C 1*s*. **g** Elemental proportions of zinc metal at different depths. ToF–SIMS of Zn anode after cycling with **h** ZW_5_G_5_M_1_ electrolyte, **i** ZSO electrolyte

In addition to reduced water coordination and selective surface adsorption, the formation of a SEI can also hinder H_2_O-induced degradation. The highest occupied molecular orbital (HOMO) and lowest unoccupied molecular orbital (LUMO) energies of MSA, GL, and H_2_O were calculated (Fig. S7). The LUMO values for MSA (1.914 eV) and GL (1.282 eV) are lower than that of H_2_O (2.205 eV), and HOMO values for MSA (− 9.485 eV) and GL (− 10.46 eV) are higher than that of H_2_O (− 11.78 eV), suggesting that MSA and GL are more easily to react and produce reaction products on the Zn surface [67]. This promotes preferential decomposition and SEI formation, providing an additional layer of protection for the Zn anode. To verify the formation of the SEI, the elemental composition of the Zn anode surface was analyzed. As shown in Fig. 3a, TEM mapping reveals that elements associated with GL and MSA are uniformly distributed across the surface of the plate-like Zn crystals. The high-resolution TEM image in Fig. 3b shows the presence of several crystalline phases on the Zn surface, including ZnCO_3_ (0.27 nm) and ZnS (0.32 nm), as highlighted in Fig. 3c, d. Furthermore, a surface layer approximately 7–10 nm thick is observed, corresponding to the SEI formed on the Zn anode. To further confirm the existence and composition of the SEI, X-ray photoelectron spectroscopy (XPS) depth profiling was performed on Zn anodes after 50 charge–discharge cycles. As shown in Fig. 3e, f, on the surface of the Zn anode, when the etching depth is 0 nm, the main source of the signal peak are the residual electrolyte and oxide, thus the characteristic peak of Zn–S is not obvious. With increasing etching depth, characteristic peaks corresponding to Zn–S, C–O, and C–N bonds become apparent, indicating the presence of SEI components. The elemental content analysis of the Zn anode at different depths was conducted via XPS, with the results shown in Fig. 3g. At the surface of the Zn metal, the Zn content accounts for only 7.63%, while the proportions of C, S, and N elements in the first layer are higher than other depths. This further confirms the presence of the SEI layer, which is composed of multiple elements. These elements originate from MSA and GL, providing evidence that MSA and GL promote the formation of SEI. Time-of-flight secondary ion mass spectrometry (TOF–SIMS) was employed to analyze the elemental distribution within the SEI. The scanned area was 100 μm × 100 μm with a depth of 100 μm. As shown in Fig. 3h, i, the surface composition of the Zn anode varied significantly depending on the electrolyte used. After 50 cycles in the ZW_5_G_5_M_1_ electrolyte, the Zn surface showed substantial enrichment of sulfur- and oxygen-containing species, along with trace amounts of carbon- and nitrogen-containing compounds. In contrast, the surface of the Zn anode cycled in the ZSO electrolyte exhibited no such aggregation, with S, O, and C elements exhibiting a much looser distribution. These results indicate that the SEI formed in the ZW_5_G_5_M_1_ electrolyte is composed primarily of S, O, C, N, and Zn species, while no significant SEI layer is formed in the ZSO electrolyte. This demonstrates that the introduction of GL and MSA facilitates the formation of a stable SEI on the Zn anode surface, effectively protecting the anode and suppressing HER. The protective effect of GL and MSA is further confirmed by Tafel curve analysis (Fig. S8). The corrosion potential of the Zn||Zn symmetric cell in the ZW_5_G_5_M_1_ electrolyte was measured at −0.022 V, significantly higher than that of the ZSO-based cell (−0.052 V), suggesting a reduced corrosion tendency in the modified electrolyte.

### Effect of GL and MSA on the Morphology of the Zn Anode

**Fig. 4:**
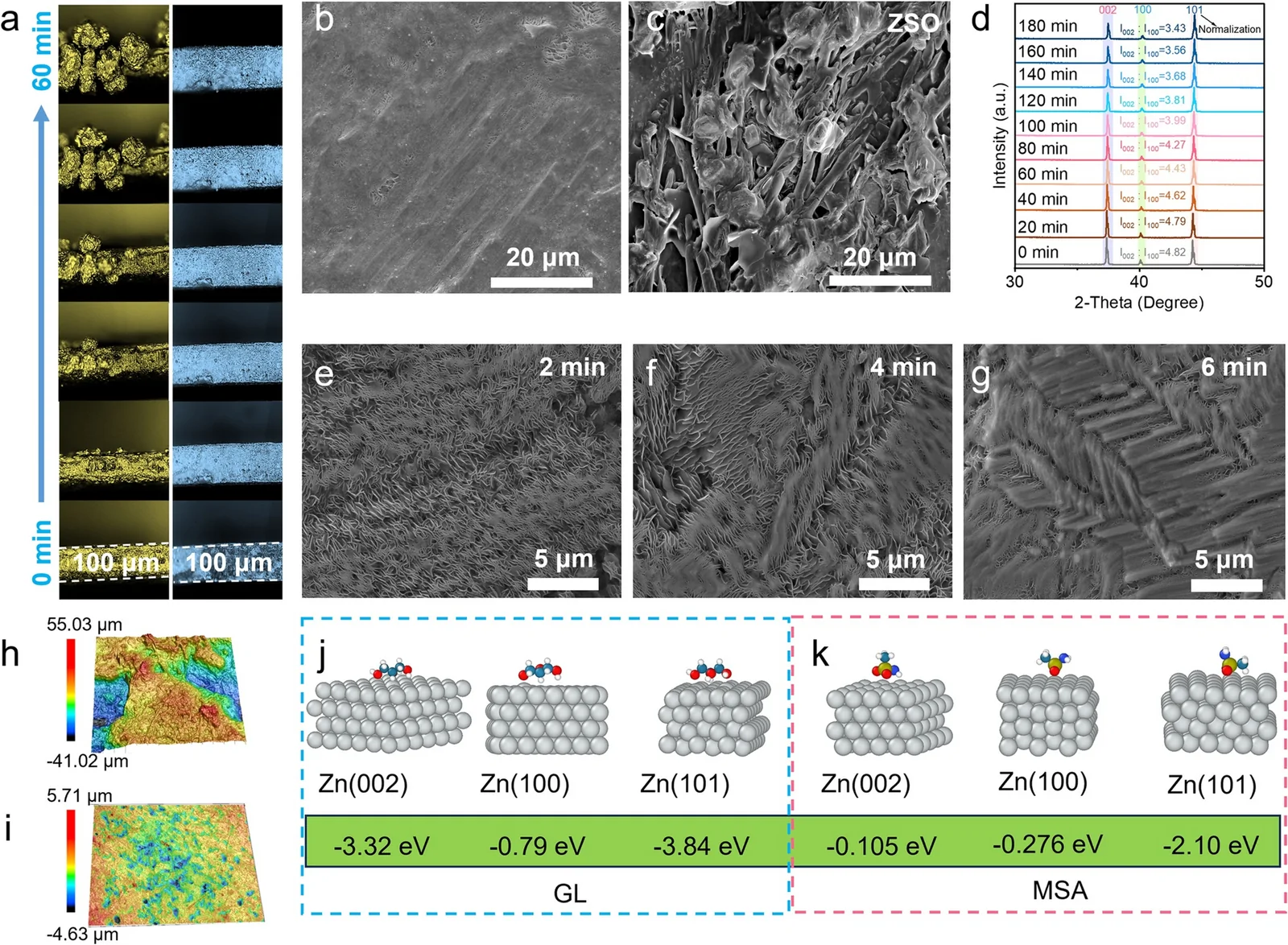
**a** In situ observation of the Zn deposition process using ZSO (left) and ZW_5_G_5_M_1_ (right) electrolytes. Zn deposited with **b** ZW_5_G_5_M_1_ electrolyte, **c** ZSO electrolyte. **d** In situ XRD of Zn deposited on Zn metal using ZW_5_G_5_M_1_ electrolyte. SEM images of Zn anode deposited for **e** 2 min, **f** 4 min, **g** 6 min using ZW_5_G_5_M_1_ electrolyte. Confocal microscopy image of the Zn foil after cycling with **h** ZSO electrolyte, **i** ZW_5_G_5_M_1_ electrolyte. The adsorption energies of **j** GL and **k** MSA on different crystal planes of Zn

Beyond mitigating side reactions, suppressing dendrite growth is also critical to extending Zn anode lifespan. Zn was electrodeposited at a current density of 10 mA cm^−2^ using different electrolytes, and the deposition process was monitored in situ via optical microscopy. As shown in Fig. 4a, extensive dendrite formation occurs on the Zn surface in the ZSO electrolyte with increasing deposition time. In contrast, the Zn surface in the ZW_5_G_5_M_1_ electrolyte thickens progressively and uniformly, maintaining a smooth, compact morphology throughout the deposition, indicating uniform Zn deposition and effective suppression of dendrites.

To gain further insight into Zn deposition behavior, scanning electron microscopy (SEM) was used to characterize the Zn surface morphology after deposition in different electrolytes. As shown in Figs. S9 and 4b, c, Zn deposited from the ZSO electrolyte exhibits dendritic agglomeration and uneven growth. Moreover, in electrolytes containing high concentrations of GL but no MSA, Zn deposition becomes increasingly non-uniform, as the elevated viscosity leads to severe concentration polarization. Reducing the GL content results in a smoother Zn surface, although the Zn deposits remain loosely packed due to irregular growth orientation. Notably, when MSA is introduced into the electrolyte, Zn deposition occurs predominantly along the (100) crystal plane, forming a dense and dendrite-free structure. These observations suggest that while GL helps suppress dendrite formation, excessive GL induces uneven Zn deposition. MSA, by contrast, guides Zn growth along the (100) orientation, facilitating vertical, compact, and uniform Zn deposition. X-ray diffraction (XRD) analysis further elucidates the effect of GL and MSA on the crystalline orientation of deposited Zn. After depositing 5 mAh cm^−2^ of Zn in various electrolytes, the intensity of different Zn crystal planes is compared. As shown in Fig. S10, increasing the GL content enhances the relative proportion of the Zn (100) plane. Upon MSA introduction, the proportion of Zn (100) initially increases and then decreases at higher concentrations. These results confirm that both GL and MSA promote Zn growth along the (100) orientation, although MSA exhibits a concentration-dependent effect on crystal plane selectivity. The crystal orientation of the Zn anode surface after cycling with ZSO and ZW_5_G_5_M_1_ electrolytes was further confirmed through a comparison of the EBSD (electron backscatter diffraction) images. As shown in Fig. S11, a significant presence of (100) crystal planes (highlighted in blue) was observed on the Zn anode surface after cycling with ZW_5_G_5_M_1_ electrolyte. In contrast, the EBSD image of the Zn anode cycled with ZSO electrolyte displayed a mixture of various crystal orientations. This further supports the conclusion that the ZW_5_G_5_M_1_ electrolyte is more favorable for guiding the formation of (100)-oriented Zn.

To balance the uniformity of Zn crystal orientation with electrolyte ionic conductivity, the ZW_5_G_5_M_1_ electrolyte was selected as the optimal formulation. To further verify that ZW_5_G_5_M_1_ promotes Zn growth along the (100) crystal plane, in situ XRD measurements were conducted on Zn||Zn symmetric cells during continuous Zn deposition at a current density of 0.2 mA cm^−2^. As shown in Fig. 4d, with increasing deposition time, the intensity of the Zn (002) peak decreases, and the intensity ratio of Zn (002) to Zn (100) also gradually diminishes, indicating an increasing proportion of Zn (100) orientation. Given that the XRD signal reflects the entire ~ 10 μm-thick Zn anode, and the amount of newly deposited Zn is relatively small, changes in the Zn (100) peak intensity are less prominent. To further explore the Zn deposition process in the ZW_5_G_5_M_1_ electrolyte, Zn was deposited at 5 mA cm^−2^ for varying durations, and the resulting morphologies were examined (Figs. S12 and 4e–g). After 2 min of deposition, vertically oriented Zn nuclei were uniformly distributed across the Zn anode surface, exhibiting an unaggregated, loosely packed morphology without dendrites. With longer deposition times, the vertically aligned Zn became more densely packed, and the surface remained smooth and compact. After 10 min of deposition, small protrusions began to appear, marking the formation of a secondary Zn layer. As deposition continued, these protrusions gradually grew but remained under 50 μm in size even after 60 min. Eventually, these protrusions evolved into a next compact Zn layer. These observations suggest that despite the vertical growth tendency of Zn, the ZW_5_G_5_M_1_ electrolyte enables uniform Zn deposition and effectively suppresses dendrite formation, even during extended cycling. Confocal microscopy was used to assess the overall topography of Zn anodes after cycling in different electrolytes. As shown in Fig. 4h, the Zn surface cycled in ZSO electrolyte exhibits pronounced unevenness, with a height variation exceeding 90 μm across a 530 μm × 700 μm area. In contrast, the Zn surface cycled in ZW_5_G_5_M_1_ (Fig. 4i) remains smooth and flat over a 530 μm × 710 μm area, with a maximum height variation of only ~ 10 μm. These results further confirm the ability of ZW_5_G_5_M_1_ to suppress dendritic growth and guide uniform Zn deposition.

To elucidate the mechanism by which GL and MSA guide Zn growth along the Zn (100) crystal plane, the adsorption energies of GL on various Zn crystal planes at different angles were calculated. As shown in Fig. 4j, GL exhibits adsorption energies of −3.32 eV on Zn (002), −0.79 eV on Zn (100), and−3.84 eV on Zn (101), indicating that GL preferentially adsorbs onto Zn (101) planes. This preferential adsorption inhibits Zn growth on the Zn (101) plane, indirectly promoting deposition along the Zn (100) orientation. Similarly, as shown in Fig. 4k, MSA exhibits adsorption energies of −0.105 eV (Zn 002), −0.276 eV (Zn 100), and −2.10 eV (Zn 101), confirming its preferential adsorption on Zn (101) and partial affinity for Zn (100). When MSA concentration is low, the Zn (101) sites are not fully saturated, allowing Zn to preferentially deposit along the (100) plane. However, at higher MSA concentrations, excess MSA also adsorbs onto the Zn (100) plane, reducing its growth rate. These findings align well with XRD observations. Enhancing battery performance at high current densities requires Zn anodes with dominant Zn (100) orientation. As reported by Fu et al., the stripping energies for Zn atoms from the Zn (002), Zn (100), and Zn (101) planes are 1.72, 1.16, and 1.24 eV, respectively, confirming that Zn (100) facilitates the fastest stripping kinetics [62]. Thus, Zn (100) is more suitable for rapid deposition/stripping under high current densities. This conclusion is further supported by CV and voltage hysteresis comparisons. As shown in Fig. S13a, for Zn||Ti asymmetric cells, decreasing GL content lowers electrolyte viscosity, thereby enhancing redox kinetics and reducing nucleation overpotential. Furthermore, at constant GL content, moderate MSA addition increases Zn (100) orientation, further reducing nucleation overpotential. A direct comparison of CV curves in ZSO and ZW_5_G_5_M_1_ (Fig. S13b) reveals that, despite the lower conductivity and higher viscosity of ZW_5_G_5_M_1_, the high reactivity of the Zn (100) plane compensates for these drawbacks, resulting in lower nucleation overpotential.

### Electrochemical Performance of Zn||Zn Symmetric Cell and Zn||Ti Asymmetric Cell

**Fig. 5:**
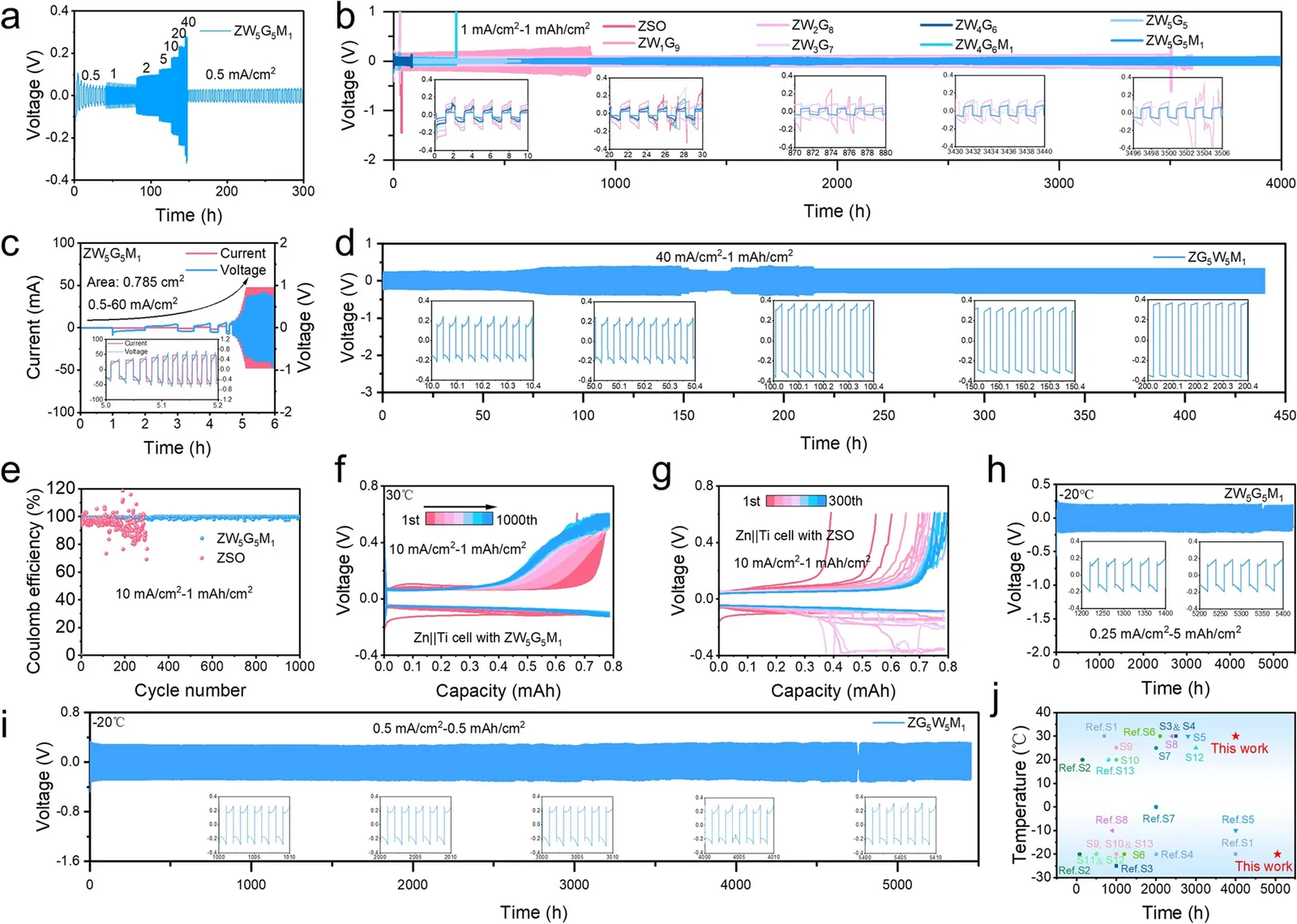
**a** Rate performance of Zn||Zn symmetric batteries assembled with ZW_5_G_5_M_1_ electrolyte. **b** Long cycle performance of Zn||Zn symmetric batteries assembled with different electrolytes. **c** CCD test of Zn||Zn symmetric batteries assembled with ZW_5_G_5_M_1_ electrolyte. **d** Long cycle performance of Zn||Zn symmetric batteries assembled with ZW_5_G_5_M_1_ electrolyte at 40 mA cm^−2^ and 1 mAh cm^−2^. **e** Coulombic efficiency of Zn||Ti asymmetric cells assembled with ZSO and ZW_5_G_5_M_1_ electrolytes. Charge–discharge curves of Zn||Ti asymmetric cells assembled with **f** ZW_5_G_5_M_1_, **g** ZSO electrolytes. Zn||Zn symmetric batteries assembled with ZW_5_G_5_M_1_ electrolyte at −20 °C with a current density of **h** 0.25 mA cm^−2^ and 5 mAh cm^−2^, **i** 0.5 mA cm^−2^ and 0.5 mAh cm^−2^. **j** Performance comparison chart of Zn||Zn symmetric batteries with different electrolytes

The suitability of Zn (100) orientation for high current densities is further validated through galvanostatic cycling tests. As shown in Fig. S14, several electrolytes containing GL could only support low current densities due to viscosity-induced limitations. However, the introduction of MSA reduces voltage hysteresis, in agreement with the CV results. As shown in Fig. 5a, the Zn||Zn symmetric cell using ZW_5_G_5_M_1_ demonstrates excellent rate capability, sustaining stable operation at 40 mA cm^−2^, whereas the ZSO-based cell fails to operate stably even at 5 mA cm^−2^. Long-term cycling tests at 1 mA cm^−2^ (1 mAh cm^−2^) in Fig. 5b reveal that GL significantly improves the Zn anode lifespan, with ZW_5_G_5_M_1_ enabling stable cycling for over 4,000 h. Due to the excellent high-rate performance of ZW_5_G_5_M_1_, critical current density (CCD) and high-current stability tests were also conducted. As shown in Fig. 5c, Zn||Zn symmetric cells in ZW_5_G_5_M_1_ operate stably at up to 60 mA cm^−2^. A constant current test at 40 mA cm^−2^ demonstrates over 400 h of stable cycling, with cumulative charge exceeding 8,500 mAh (Fig. 5d). These findings confirm that ZW_5_G_5_M_1_ enables stable Zn deposition/stripping even under high-current conditions, providing a practical route toward fast-charging AZIBs.

In general, side reactions significantly compromise the reversibility of Zn anodes. To assess the effect of the ZW_5_G_5_M_1_ electrolyte on Zn reversibility, deposition–stripping tests were conducted using Zn||Ti asymmetric cells. As shown in Fig. 5e–g, at a current density of 10 mA cm^−2^ and a capacity of 1 mAh cm^−2^, the Zn||Ti asymmetric cell assembled with the ZSO electrolyte exhibited rapid performance degradation and failed within 200 cycles. In stark contrast, the Zn||Ti asymmetric cell using the ZW_5_G_5_M_1_ electrolyte maintains a high coulombic efficiency of 98.7% over 1,000 deposition–stripping cycles, confirming the superior reversibility of Zn in the ZW_5_G_5_M_1_ system. As discussed earlier, the incorporation of GL and MSA effectively restructures the hydrogen-bond network within the electrolyte. The resulting increase in entropy helps to prevent electrolyte condensation, particularly at low temperatures. To verify this effect, galvanostatic charge–discharge tests were performed on Zn||Zn symmetric cells assembled with ZSO and ZW_5_G_5_M_1_ electrolytes at −20 °C. As presented in Fig. S15 the Zn||Zn symmetric cell with ZW_5_G_5_M_1_ demonstrates a wide low-temperature window from 0 to -40 °C under 0.25 mA cm^−2^ and 0.25 mAh cm^−2^. Moreover, as presented in Figs. 5h, i and S16, the Zn||Zn symmetric cell with ZW_5_G_5_M_1_ demonstrates excellent low-temperature stability, sustaining over 5,400 h of cycling at 0.5 mA cm^−2^ and 0.5 mAh cm^−2^, over 5,000 h at 0.25 mA cm^−2^ and 0.25 mAh cm^−2^, and over 5,400 h at 0.25 mA cm^−2^ and 5 mAh cm^−2^. In comparison, as shown in Fig. S17, the cell with the ZSO electrolyte failed to operate under the same low-temperature conditions. The CCD testing of Zn||Zn symmetric cells at −20 °C further investigates the reversibility and current density range of the battery at low temperatures. As shown in Fig. S18 unlike the failure observed in Zn||Zn symmetric cells with ZSO electrolyte, the Zn||Zn symmetric cells with ZW_5_G_5_M_1_ electrolyte were able to operate stably within a current density range of 0.1–25 mA cm^−2^ and then return to stable operation at 0.1 mA cm^−2^ after the test. This indicates that the ZW_5_G_5_M_1_ electrolyte enables the Zn anode to exhibit excellent reversibility at low temperature. In addition, as shown in Fig. S19, under 0.25 mA cm^−2^ and 0.25 mAh cm^−2^, the Zn||Ti asymmetric cells exhibited a coulombic efficiency of 99.1% after 1,200 cycles of Zn deposition and stripping at −20 °C, demonstrating that batteries assembled with the ZW_5_G_5_M_1_ electrolyte maintain high Zn reversibility even at low temperatures. These results conclusively demonstrate that the combined addition of GL and MSA substantially broadens the operational temperature window of the electrolyte, enabling stable Zn electrochemistry even at sub-zero temperatures.

### Electrochemical Performance of Zn||VO_2_ Full Cell

**Fig. 6:**
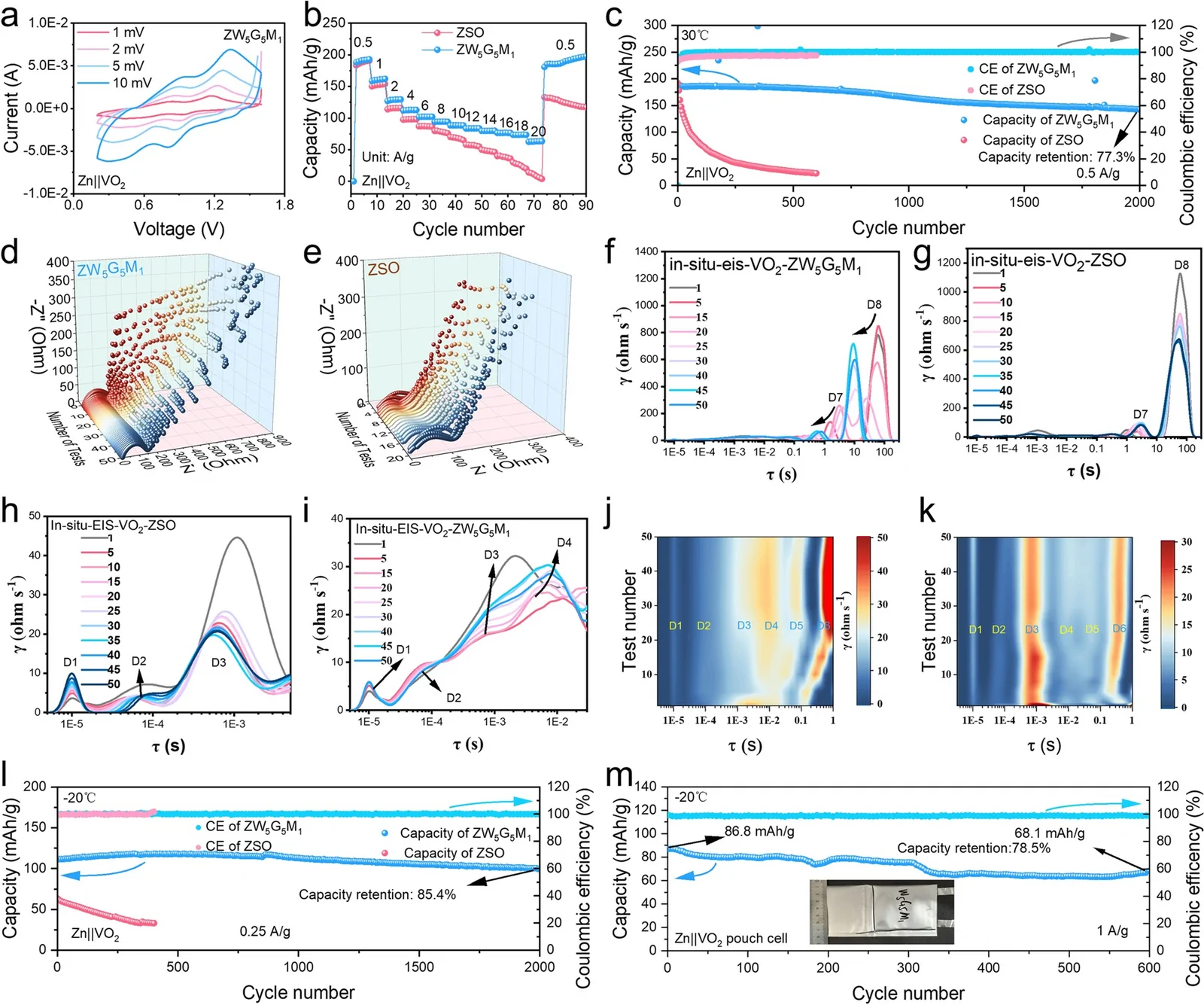
**a** CV curves of the Zn||VO_2_ full cells assembled with ZW_5_G_5_M_1_ electrolyte. **b** Rate performance of the Zn||VO_2_ full cells. **c** Long cycle performance of the Zn||VO_2_ full cells. In situ impedance testing of the Zn||VO_2_ full cells assembled with **d** ZW_5_G_5_M_1_ electrolyte and **e** ZSO electrolyte. DRT curves of Zn||VO_2_ full cell with **f** and **i** ZW_5_G_5_M_1_, **g** and **h** ZSO electrolytes. DRT contour map of Zn||VO_2_ full cell with **j** ZW_5_G_5_M_1_ and **k** ZSO electrolytes. Long-cycle performance of **l** the Zn||VO_2_ full cell and **m** the Zn||VO_2_ pouch cell assembled with ZW_5_G_5_M_1_ electrolyte at −20 °C

Zn||VO_2_ full cells were assembled using ZSO and ZW_5_G_5_M_1_ electrolytes to evaluate their electrochemical performance. As shown in Figs. 6a and S20, the cyclic voltammetry (CV) curves of both electrolytes exhibit similar peak positions and quantities, indicating that the introduction of the ZW_5_G_5_M_1_ electrolyte does not introduce any additional redox reactions. In the rate performance test shown in Fig. 6b, both cells demonstrate comparable initial capacities. However, with continued cycling, the full cell with the ZSO electrolyte exhibits significant capacity degradation, while the cell with the ZW_5_G_5_M_1_ electrolyte maintains a stable capacity across a wide range of current densities (0.5–20 A g^−1^), underscoring its excellent rate performance and practical applicability. In the long-term cycling tests under constant current conditions, the full cell with the ZW_5_G_5_M_1_ electrolyte exhibits outstanding capacity retention. As shown in Fig. 6c, at 0.5 A g^−1^, the full cell with the ZSO electrolyte experiences rapid capacity decay, retaining only 12.5% of its initial capacity after 600 cycles. In contrast, the full cell with the ZW_5_G_5_M_1_ electrolyte retains a capacity of 143.6 mAh g^−1^ after 2,000 cycles, corresponding to a capacity retention of 77.3%. The charge–discharge curves presented in Fig. S21 further confirm that the full cell with the ZW_5_G_5_M_1_ electrolyte exhibits negligible capacity fading after 2,000 cycles, highlighting the electrolyte’s stability and durability.

In situ EIS tests were also performed to monitor the interfacial resistance evolution during cycling. As shown in Fig. 6d, since the ionic conductivity of ZW_5_G_5_M_1_ electrolyte is lower than that of ZSO electrolyte, the full cell with ZW_5_G_5_M_1_ electrolyte exhibits greater resistance. It is worth noting that the diameter of the semicircle and the slope of the low-frequency region initially decrease and then stabilize with cycling. This behavior suggests an initial acceleration of the electrochemical kinetics, likely due to electrolyte infiltration and electrode activation, followed by the formation of a stable SEI that moderately increases charge transfer resistance before reaching equilibrium. In contrast, the EIS curve of the cell with ZSO electrolyte shows a semicircular region that first decreases and then increases. The initial decrease in the semicircle is likely due to the electrolyte infiltration, which improves the contact between the electrode and electrolyte. The subsequent increase may be caused by side reactions that affect the reversibility of the battery.

The EIS curve still has certain limitations in impedance analysis. To more accurately investigate the contribution and variation of each reaction process to the impedance, we performed DRT analysis on the in situ EIS results, with the relevant results shown in Figs. 6f–k and S22. In DRT curves, D1 corresponds to the electronic relaxation related to the contact resistance between the current collector and the electrode interface, as well as between electrode particles. D2 corresponds to the absorption and desolvation process of the solvation structure at the interface. The D3 peak appears in the time constant range of approximately 10^−3^ s, which is typically associated with the ion transport process at the SEI in the battery. The intensities of the D4 and D5 peaks are closely related to the migration of Zn^2+^/Zn⁺/Zn on the Zn surface and the subsequent formation of Zn crystals. We attribute the D6 peak to the charge transfer process of Zn^2+^ at the interface, while the D7 and D8 peaks are related to the diffusion processes of Zn^2+^ and hydrated zinc ions [68]. As shown in Fig. 6h, the D2 peak of the Zn||VO_2_ battery using ZSO electrolyte is lower, which is due to the low viscosity of ZSO electrolyte, which facilitates the migration of the hydrated layer, resulting in a lower impedance in the D2 region. In contrast, as shown in Fig. 6i, in the higher viscosity ZW_5_G_5_M_1_ electrolyte, the migration of the Zn hydrated layer is more difficult, leading to a higher impedance in the D2 region. Moreover, in the ZW_5_G_5_M_1_ electrolyte, the D3 region shows a gradual increase followed by stabilization, with the increase in impedance primarily attributed to the formation of the SEI. As the reaction progresses, the SEI stabilizes, and the impedance change becomes more stable. In contrast, in the ZSO electrolyte, the changes in the D3 region are characterized by irregular fluctuations, which proves that the electrode interface of ZSO is unstable.

As shown in Fig. S22a, b, after the introduction of GL and MSA, the peak value in the D4–D8 region is higher than that observed in the ZSO electrolyte without MSA and GL. This is because, in ZSO electrolyte, the migration of Zn^2+^/Zn^+^/Zn is more rapid, leading to a lower peak intensity in the D4 region. At the same time, high viscosity, crystal plane shielding, and low moisture double layers slow down the migration of Zn^2+^, resulting in overall higher DRT values. As shown in Fig. 6j, k, unlike the regular increase in DRT values observed in the battery with ZW_5_G_5_M_1_ electrolyte, the DRT of the battery assembled with ZSO electrolyte exhibits fluctuating changes in multiple regions (such as D3–D6). This may be due to the susceptibility of the ZSO electrolyte to side reactions.

Given the demonstrated stability of the ZW_5_G_5_M_1_ electrolyte at −20 °C, galvanostatic cycling tests were performed at −20 °C (Fig. 6l). At a current density of 0.25 A g^−1^, the full cell with the ZW_5_G_5_M_1_ electrolyte retains a capacity of over 100 mAh g^−1^ after 2,000 cycles, achieving a high capacity retention of 85.4%. In contrast, the full cell with the ZSO electrolyte exhibits almost complete capacity loss due to electrolyte freezing. As shown in Fig. S23, despite the increased polarization at low temperatures, the full cell with the ZW_5_G_5_M_1_ electrolyte maintains normal charge–discharge behavior, confirming its functionality under low-temperature conditions. To further assess the practical viability of the ZW_5_G_5_M_1_ electrolyte, a Zn||VO_2_ pouch cell with the ZW_5_G_5_M_1_ electrolyte was assembled and tested. As shown in Fig. 6m, the pouch cell exhibits excellent long cycle stability, retaining 68.1 mAh g^−1^ after 600 cycles at 1 A g^−1^, corresponding to a capacity retention of 78.5%. The corresponding charge–discharge curves (Fig. S24) remain stable, with no new voltage plateaus observed, further confirming that the ZW_5_G_5_M_1_ electrolyte supports stable cycling without inducing side reactions. These results strongly demonstrate the suitability and scalability of ZW_5_G_5_M_1_ for use in practical aqueous zinc-ion battery applications.

## Conclusion

In this study, glycerol (GL) and methylsulfonamide (MSA) were incorporated into the ZSO electrolyte to reconstruct the hydrogen-bond network and suppress the activity of free water molecules, thereby effectively increasing the configurational entropy (*S*_*conf*_) of the electrolyte and lowering its freezing point. The selective adsorption of GL and MSA molecules on specific Zn crystal planes occupied preferential nucleation sites, synergistically guiding Zn growth along the Zn (100) crystal plane. This facilitated uniform, vertically aligned, dendrite-free Zn deposition and enables the construction of a highly active Zn anode. Additionally, GL molecules adsorbed onto the Zn surface to form a protective layer that mitigates the direct attack of free water molecules, while the solid electrolyte interphase layer formed by GL and MSA further suppresses parasitic side reactions. As a result, Zn||Zn symmetric cells exhibited excellent electrochemical performance, achieving stable cycling for 4,000 h of cycling at 1 mA cm^−2^, and 600 h at 40 mA cm^−2^ under a capacity of 1 mAh cm^−2^. Surprisingly, the symmetric cells maintained over a staggering 5,400 h of stable cycling even at the extreme −20 °C. Furthermore, Zn||VO_2_ full cells with the electrolyte demonstrated remarkable capacity retention of 77.3% after 2,000 cycles at 30 °C and 0.5 A g^−1^, and a remarkable capacity retention of 85.4% after 2,000 cycles even at −20 °C and 0.25 A g^−1^. These findings clearly demonstrate that the proposed electrolyte design effectively broadens both the current and temperature operating windows of AZIBs, offering a promising strategy for advancing their practical and diversified applications.

## Supplementary Information

Below is the link to the electronic supplementary material.

Supplementary file1 (DOCX 31495 KB)
