# Supplementary material for: Decoding Hydrogen-Bond Network of Electrolyte for Cryogenic Durable Aqueous Zinc-Ion Batteries
Source: Nanomicro Lett. 2026 Jan 3;18:127. doi: 10.1007/s40820-025-01970-3 (PMC12759032; doi:10.1007/s40820-025-01970-3)
Supplement: Supplementary file 1 — Supplementary file1 (DOCX 31495 KB) [file 40820_2025_1970_MOESM1_ESM.docx]

Supporting Information for

**Decoding Hydrogen-Bond Network of Electrolyte for Cryogenic Durable Aqueous Zinc-Ion Batteries**

Xiyan Wei^1,2,4^#, Jinpeng Guan^1,2,4^#, Yongbiao Mu^1,2^*, Yuhan Zou^3^, Xianbin Wei^5^, Lin Yang^1,2^, Quanyan Man^1,2^, Chao Yang^4^, Limin Zang^4^*, Jingyu Sun^3^*, and Lin Zeng^1,2^*

^1^Shenzhen Key Laboratory of Advanced Energy Storage, Department of Mechanical and Energy Engineering, Southern University of Science and Technology, Shenzhen 518055, P. R. China

^2^SUSTech Energy Institute for Carbon Neutrality, Southern University of Science and Technology, Shenzhen 518055, P. R. China

^3^College of Energy, Soochow Institute for Energy and Materials Innovations, Key Laboratory of Advanced Carbon Materials and Wearable Energy Technologies of Jiangsu Province, Soochow University, Suzhou 215006, P. R. China

^4^MOE Key Laboratory of New Processing Technology for Nonferrous Metal and Materials, Key Laboratory of Natural and Biomedical Polymer Materials (Education Department of Guangxi Zhuang Autonomous Region), College of Materials science and Engineering, Guilin University of Technology, Guilin 541004, P. R. China

^5^Department of Materials Science and Engineering, Southern University of Science and Technology, Shenzhen 518055, P. R. China

# Xiyan Wei and Jinpeng Guan contributed equally to this work.

*Corresponding authors. E-mail: [muyb2021@mail.sustech.edu.cn](mailto:muyb2021@mail.sustech.edu.cn) (Yongbiao Mu); [2016034@glut.edu.cn](mailto:2016034@glut.edu.cn) (Limin Zang); [sunjy86@suda.edu.cn](mailto:sunjy86@suda.edu.cn) (Jingyu Sun); [zengl3@sustech.edu.cn](mailto:zengl3@sustech.edu.cn) (Lin Zeng)

**S1 Experimental Section**

**S1.1 Materials**

Zinc (Zn) foils (99.99%, 10 µm and 100 µm thickness) and titanium (Ti) foils (99.99%, 10 µm thickness) were purchased from Shenzhen Kejing Star Technology Co., Ltd. N-methyl-2-pyrrolidone (NMP), vanadium dioxide (VO_2_), and zinc sulfate heptahydrate (ZnSO_4_·7H_2_O) were obtained from Shanghai Macklin Biochemical Co., Ltd. Super P conductive carbon black was supplied by Jiangsu Shenzhou Carbon Co., Ltd. Methylsulfonamide (MSA) and glycerol (GL) were procured from Shanghai Aladdin Biochemical Technology Co., Ltd. All chemicals and materials used were of analytical grade and were utilized without any further purification.

**S1.2 Electrolyte Preparation**

The 2 M ZnSO_4_ (ZSO) electrolyte was prepared by dissolving ZnSO_4_·7H_2_O in deionized water using a 100 mL volumetric flask. The ZW_a_G_b_M_c_ electrolytes were formulated by mixing ZnSO_4_·7H_2_O, deionized water, GL, and MSA. Initially, GL and deionized water were combined in volume ratios of 1:9, 2:8, 3:7, 4:6, and 5:5. Subsequently, ZnSO_4_·7H_2_O and MSA at various concentrations were added to the mixture, followed by continuous stirring until a homogeneous electrolyte solution was obtained.

**S1.3 Materials Characterizations**

The morphologies of the samples were examined using a Hitachi SU-8230 field-emission scanning electron microscope (FE-SEM). Transmission electron microscopy (TEM), energy-dispersive X-ray spectroscopy (EDX), and elemental mapping were carried out on a Thermo Fisher Talos microscope operated at an acceleration voltage of 300 kV. X-ray photoelectron spectroscopy (XPS) was conducted using a Thermo Scientific ESCALAB 250Xi system with an Al Kα radiation source (hν=1,486.8 eV). Raman spectra were collected using a HORIBA LabRAM HR Evolution spectrometer with a 532 nm laser as the excitation source. X-ray diffraction (XRD) patterns were recorded on a Bruker D8 Advance diffractometer equipped with a D/Tex Ultra detector and Cu-Kα radiation, operated at a scan rate of 5°/min, to analyze the crystalline structure of the samples.

**S1.4 *In Situ* Optical Microscope Characterization**

The cells for in situ optical microscopy observations were assembled using molds purchased from Beijing Scistar Technology Co., Ltd. Both the working and counter electrodes consisted of Zn metal. The electrolytes employed were ZSO and ZW_5_G_5_M_1_ An electrochemical workstation was used to supply the power, with a constant current density of 10 mA/cm^2^ applied during the experiments.

**S1.5 Electrochemical Performance Assessment**

Zn||Zn symmetric cells were assembled by sandwiching a glass fiber separator (Whatman GF/D) between commercial Zn plates (10 mm in diameter) in CR2025-type coin cells, which were filled with 70 μL of the respective electrolytes. Zn||Ti half-cells were assembled using Zn plates (10 mm in diameter) as the anode, Ti foils (16 mm in diameter) as the cathode, and GF/D glass fiber as separators. These cells were also assembled in CR2025-type coin cells and filled with 70 μL of different electrolytes. Zn||VO_2_ full cells were fabricated using Zn plates (14 mm in diameter) as the anode and VO_2_ electrodes (12 mm in diameter) as the cathode, with 80 μL of either ZSO or ZW_5_G_5_M_1_ electrolytes. The VO_2_ cathode was prepared by mixing VO_2_, Super P carbon black, and PVDF binder in a mass ratio of 8:1:1, using NMP as the solvent. The slurry was stirred for 12 h, cast onto Ti foil, and dried at 70℃. The VO_2_ loading was ranged from 0.5 mg to 1.5 mg per electrode.

For pouch cell assembly, Zn plates (40 mm × 30 mm) were used as the anode, and VO_2_ electrodes (25 mm × 35 mm) were used as the cathode. The pouch cells were filled with 300 μL of ZW_5_G_5_M_1_ electrolyte. Nickel strips were attached to both electrodes using conductive tape. The cell stack, consisting of cathode, separator, and anode, was enclosed in an aluminum-plastic film, which was heat-sealed to ensure proper encapsulation. An additional 300 μL of electrolyte was injected into the sealed cell.

To evaluate the electrochemical performance and coulombic efficiency (CE) of Zn plating/stripping, Zn||Ti asymmetric cells were tested using a Neware battery test system (Shenzhen, China) at 30℃ with ZSO and ZW_5_G_5_M_1_ electrolytes. For CE measurements, 1 mAh/cm^2^ of Zn was plated onto Ti foil and subsequently stripped to 0.6 V in each cycle under current densities of 10 mA/cm^2^.

Cycling stability and voltage hysteresis were evaluated using Zn||Zn symmetric cells tested at various current densities (1-60 mA/cm^2^) with a fixed Zn deposition capacity of 0.25-5 mAh/cm^2^. Electrochemical impedance spectroscopy (EIS) was performed on a CHI760d electrochemical workstation (CH Instruments, Shanghai, China) over a frequency range of 100 kHz to 100 mHz.

Chronoamperometry (CA) measurements were conducted under a fixed overpotential of 0.15 V. Cyclic voltammetry (CV) tests were performed on Zn||Ti cells in both ZSO and ZW_5_G_5_M_1_ electrolytes over a voltage range of 1 V to -0.3 V at a scan rate of 1 mV/s. Linear sweep voltammetry (LSV) was carried out using Zn||Zn symmetric cells in ZSO and ZW_5_G_5_M_1_ electrolytes at a scan rate of 1 mV/s.

**S1.6 Related calculations method**

We calculated the ionic conductivity of the electrolyte using Equation S1:

$$\begin{aligned} \delta=\frac{L}{R_{S}A}\#\left( S1 \right) \end{aligned}$$

where $\delta$ represents the ionic conductivity, Rs signifies the electrolyte impedance, A denotes the contact area of the Zn sheet (diameter is 10 mm), and L indicates the distance of the separator (210 μm) immersed in the electrolytes.

Utilizing the Arrhenius equation (Equation S2) to calculate the activation energy:

$$\begin{aligned} \frac{1}{R_{ct}}=Ae^{-\frac{E_{a}}{RT}}\#\left( S2 \right) \end{aligned}$$

where Rct, A, R, and T denote the charge transfer impedance, frequency factor, gas constant, and absolute temperature respectively.

The second law of thermodynamics:

$$\begin{aligned} \Delta G=\Delta H-T\Delta S\#(S3) \end{aligned}$$

where ∆H, T, ∆S denote the enthalpy, temperature, and entropy of the system respectively.

The Equation to calculate the configurational entropy (*S_conf_*):

$$\begin{aligned} S_{conf}=-R\sum_{i=1}^{n} x_{i}lnx_{i}\#\left（ S4 \right） \end{aligned}$$

where R is the ideal gas constant, and *x_i_* is the molar fraction of the *i*th component**.**

**S1.7 DFT and MD calculation method**

Classical molecular dynamics (MD) simulations were conducted to investigate the mixed electrolytes at the atomic scale. One bulk model (System1) was constructed for these simulations. System1 comprises 200 Zn^2+^ ions, 200 SO₄^2-^ ions, 100 methylsulfonamide (MSA) molecules, and 431 glycerol (GL) molecules. The initial configuration of the system was generated using the PACKMOL software, with all species randomly placed within a cubic simulation box.

The partial charges of all molecules were calculated using the Gaussian 16 software package, employing the 6-311G (d,p) basis set. Due to the high concentration of zinc sulfate in the system, the charges of the Zn^2+^ and SO₄^2-^ ions were scaled by a factor of 0.8 to account for overestimated electrostatic interactions.

The OPLS-AA force field was applied to describe the interactions of ZnSO_4_ and the target organic molecules, while the TIP3P model was used for water molecules. The molecular force field includes both bonded and non-bonded interactions. The non-bonded interactions consist of van der Waals (vdW) forces and electrostatic interactions, which are represented by Equations (S5) and (S6), respectively.

$$\begin{aligned} E_{LJ}\left( r_{ij} \right)=4\varepsilon_{ij}\left( \left( \frac{\text{σ}_{ij}}{r_{ij}} \right)^{12}-\left( \frac{\text{σ}_{ij}}{r_{ij}} \right)^{6} \right)\#\left( S5 \right) \end{aligned}$$

$$\begin{aligned} E_{c}\left( r_{ij} \right)=\frac{q_{i}q_{j}}{4\pi\varepsilon_{o}\varepsilon_{r}r_{ij}}\#\left( S6 \right) \end{aligned}$$

In the equation, $q_{i}$、$q_{j}$ are atomic charge, $r_{ij}$is the distance between atoms, $\text{σ}$ is the atomic diameter, $\varepsilon$ is the atomic energy parameter.

For different kinds of atoms, the Lorentz-Berthelot mix rules were adopted for vdW interactions, which follows the Equation 4. The cutoff distance of vdW and electronic interactions was set to 1.2 nm, and the PME method was employed to calculate long-range electrostatic interactions.

$$\begin{aligned} \sigma_{ij}=\frac{1}{2\left( \sigma_{ii}+\sigma_{jj} \right)};\varepsilon_{ij}=\left( \varepsilon_{ii}*\varepsilon_{jj} \right)^{\frac{1}{2}}\#\left( S7 \right) \end{aligned}$$

For the MD simulations, energy minimization was first conducted to relax the initial structure of the simulation box. Subsequently, an isothermal-isobaric (NPT) ensemble was employed with a time step of 1.0 fs to further optimize the simulation box, maintaining the temperature at 298.15 K and the pressure at 1.0 atm. Temperature and pressure were regulated using the Nosé-Hoover thermostat and the Parrinello-Rahman barostat, respectively. The duration of the NPT equilibration was set to 20.0 ns, which is sufficient to achieve a stable box size.Throughout all MD simulations, atomic motion was governed by classical Newtonian mechanics, and the equations of motion were integrated using the velocity-Verlet algorithm. All MD simulations were performed using the GROMACS 2021.5 software package.

Density functional theory (DFT) calculations for water, salt, and target molecules were carried out using the Gaussian software. Geometry optimizations were conducted using the PBE1PBE functional. Implicit solvation effects were accounted for using the solvation model based on density (SMD). Molecular structures were visualized with Visual Molecular Dynamics (VMD). The binding energy (𝐸_Binding_) of the complexes was calculated according to the following equation:

$$\begin{aligned} E_{Binding}=E_{complexe1-complexe2}-E_{complexe1}-E_{complexe2}\#\left( S8 \right) \end{aligned}$$

where$E_{complexe1-complexe2}$ represents the total energy of the $complexe1$ interacting $complexe2$. $E_{complexe1}$ is the energy of the $complexe1$, and $E_{complexe2}$ is the energy of the $complexe2$.

**S1.8 Vienna ab initio Simulation (VASP) calculation method**

Unless otherwise specified, all quantum chemical calculations were performed using the Gaussian 16 A.03 software package. The geometries of all molecules were fully optimized at the B3LYP [S1] level of theory with Grimme’s D3 (BJ) empirical dispersion correction, denoted as B3LYP-D3 (BJ), in conjunction with the 6-311+G** basis set. Frequency analyses were conducted at the same level of theory to confirm the nature of the stationary points and to ensure the absence of imaginary frequencies, thereby validating the optimized structures as true minima. All geometry optimizations were carried out in the aqueous phase using the polarizable continuum model (PCM) without imposing any structural constraints.

Binding energy (ΔE) calculations were performed at the PWPB95-D3(BJ)/def2-QZVPP level using the ORCA 5.0.4 software package, incorporating the solvation model based on solute electron density (SMD) to account for solvent effects in water. Wavefunction analyses were conducted using the Multiwfn program. Selected isosurface maps were visualized with the Visual Molecular Dynamics (VMD) software, based on output files generated by Multiwfn.

**Supplementary Figures and Tables**

**Fig. S1** The electrostatic potential **a**) MSA and **b**) GL


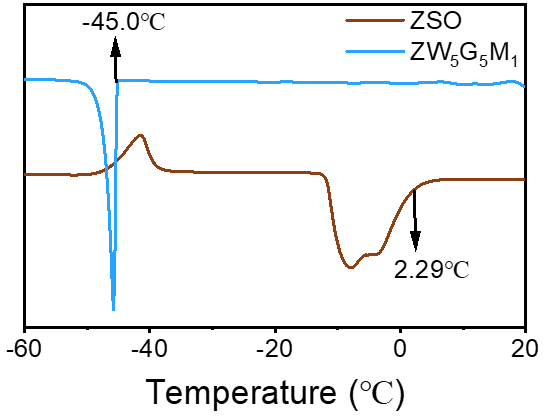


**Fig. S2** DSC of different electrolytes


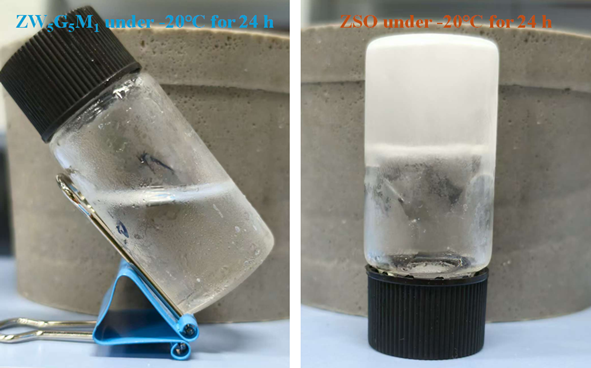


**Fig. S3** The optical images of electrolytes after 24 h of standing at -20°C

**Fig. S4** Adsorption energies of **a**) GL, **b**) MSA, and **c**) H_2_O on the Zn (101) crystal surface

**Fig. S5** Coordination numbers of components in the Zn^2+^ solvation structure

**Fig. S6** Impedance of SS||SS cells assembled with ZW_5_G_5_M_1_ and ZSO electrolytes at different temperatures


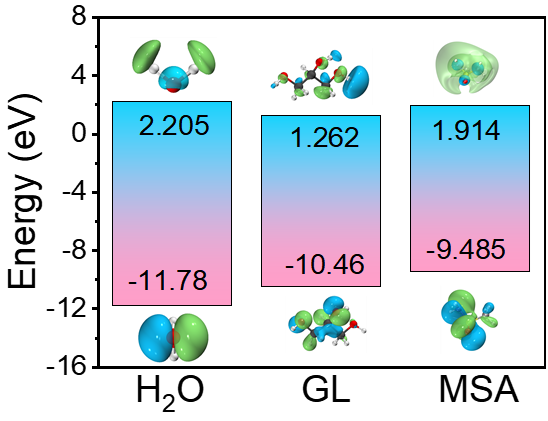


**Fig. S7** HOMO and LUMO values of H_2_O, GL, and MSA

**Fig. S8** Tafel of Zn||Zn symmetric cells assembled with different electrolytes


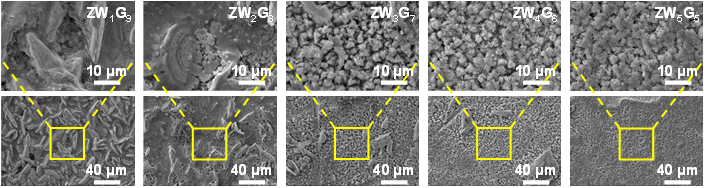


**Fig. S9** SEM of Zn deposited in electrolytes with different GL contents


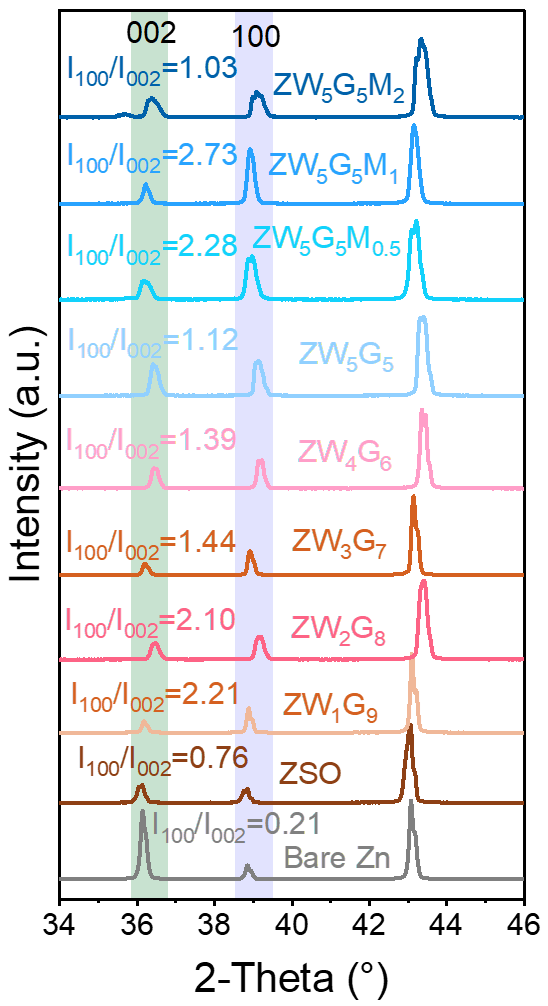


**Fig. S10** XRD of Zn anode after cycling with different electrolytes


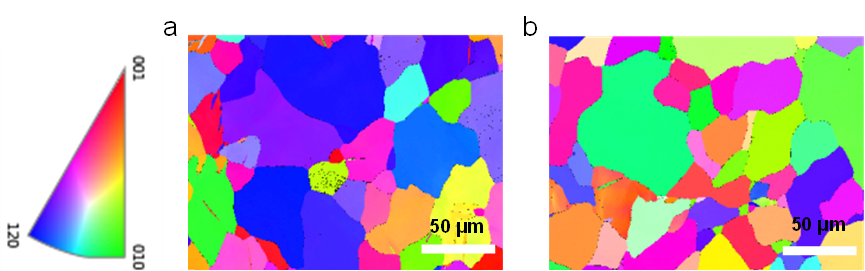


**Fig. S11** EBSD image of Zn anode after cycling with (**a**) ZW_5_G_5_M_1_, (**b**) ZSO electrolyte

**Fig. S12** **a**) High magnification and **b**) low magnification SEM of Zn anode deposited for different times in ZW_5_G_5_M_1_ electrolyte

**Fig. S13** **a**) CV curves of Zn||Ti cells assembled with different electrolytes. **b**) CV curves of Zn||Ti batteries assembled with ZSO and ZW_5_G_5_M_1_ electrolytes

**Fig. S14** Rate performance of Zn||Zn symmetric cells assembled with different electrolytes

**Fig. S15** Rate performance of Zn||Zn symmetric cells assembled with ZW_5_G_5_M_1_ electrolyte under different temperature

**Fig. S16** The long cycle performance of Zn||Zn symmetric cell at -20℃ with current densities of 0.25 mA/cm^2^ and 0.25 mAh/cm^2^


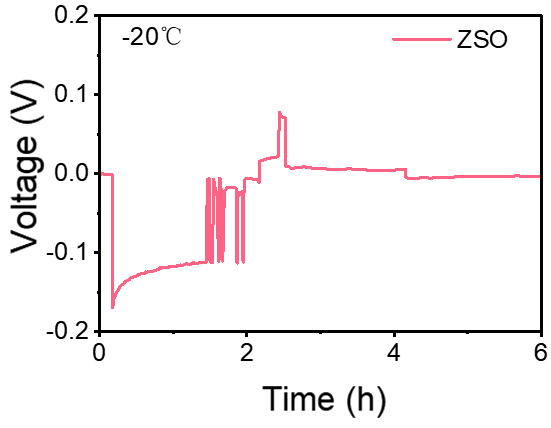


**Fig. S17** Zn||Zn symmetric cell assembled with ZSO electrolyte cannot operate at -20 ℃

**Fig. S18** CCD test of Zn||Zn symmetric cell with ZW5G5M1 electrolyte at -20 ℃

**Fig. S19 a**) Coulombic efficiency of Zn||Ti batteries assembled with ZW_5_G_5_M_1_ electrolyte at -20°C. **b**) Charge-discharge curves of Zn||Ti batteries assembled with ZW_5_G_5_M_1_ electrolyte at -20 °C

**Fig. S20** CV curves of Zn||VO_2_ batteries assembled with ZSO electrolyte

**Fig. S21** Charge-discharge curves of Zn||VO_2_ full cells assembled with ZW_5_G_5_M_1_ electrolyte


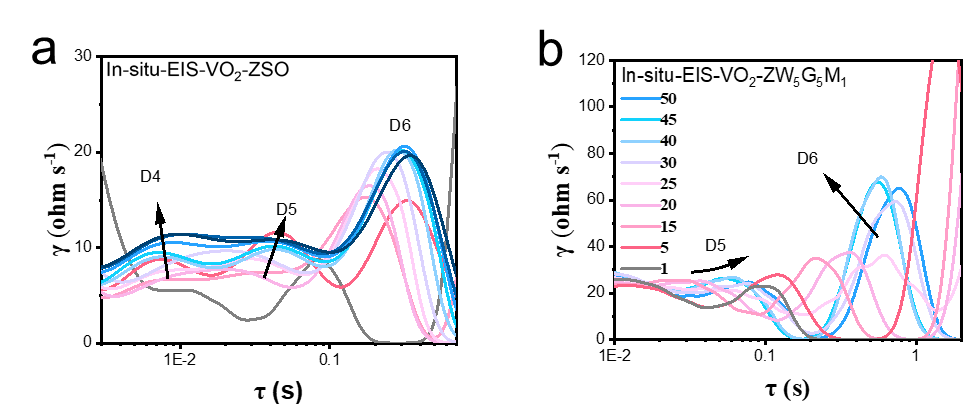


**Fig. S22** DRT curves of Zn||VO_2_ cells assembled with **a-b**) ZSO and c-d) ZW_5_G_5_M_1_ electrolytes

**Fig. S23** Charge-discharge curves of Zn||VO_2_ full cells assembled with **a**) ZW_5_G_5_M_1_ electrolyte and **b**) ZSO electrolyte

**Fig. S24** Charge-discharge curves of Zn||VO_2_ full cells assembled with **a**) ZSO electrolyte and **b**) ZW_5_G_5_M_1_ electrolyte

**Table S1** Performance comparison of Zn||Zn symmetric cells assembled with different electrolytes

| Electrolyte | Temperature (℃) | Current (mA/cm^2^) | Time (h) | Refs. |
| --- | --- | --- | --- | --- |
| ZSO+GL+MSA | 30 | 1 | 4,000 | This work |
|  | -20 | 0.5 | 5,400 |  |
| L-cysteine+ZSO | -20 | 1 | 4,000 | [S2] |
|  | 30 | 5 | 700 |  |
| ethylene glycol+ZSO | -20 | 2 | 80 | [S3] |
|  | 20 | 2 | 150 |  |
| γ-valerolactone+Zn(OTf)_2_ | 30 | 1 | 2,500 | [S4] |
|  | -25 | 0.5 | 1,000 |  |
| N,N-dimethylformamide+ZSO | 30 | 1 | 2,500 | [S5] |
|  | -20 | 0.5 | 2,000 |  |
| Tetrahydrofuran+ Zn(OTf)_2_ | 30 | 1 | 2,800 | [S6] |
|  | -10 | 1 | 4,000 |  |
| ZSO+DMSO | 30 | 1 | 2,100 | [S7] |
|  | -20 | 0.5 | 1,200 |  |
| Zn(OTf)_2_+PEGDME | 25 | 1 | 2,000 | [S8] |
|  | 0 | 1 | 2,000 |  |
| ZSO+CH_3_COONH_4_ | 30 | 2 | 2,400 | [S9] |
|  | -10 | 0.5 | 900 |  |
| Zn(ClO4)_2_+ACN | 25 | 1 | 1,000 | [S10] |
|  | -20 | 1 | 1,000 |  |
| Zn(OTf)_2_+PEG | 20 | 1 | 1,000 | [S11] |
|  | -20 | 0.2 | 1,000 |  |
| Zn(OTf)_2_/H_2_O-AN | -20 | 1 | 500 | [S12] |
| ZS0+GL+AN | 25 | 0.2 | 3,000 | [S13] |
|  | -20 | 0.2 | 500 |  |
| 1,3-dioxolane+ ZnCl_2_ | 20 | 1 | 800 | [S14] |
|  | -20 | 5 | 1,000 |  |

**Supplementary References**

1. P.J. Stephens, F.J. Devlin, C.F. Chabalowski, M.J. Frisch, Ab initio calculation of vibrational absorption and circular dichroism spectra using density functional force fields. J. Phys. Chem. **98**(45), 11623–11627 (1994). <https://doi.org/10.1021/j100096a001>
2. W. Song, X. Xie, L. Deng, A. Pan, G. Cao et al., Reversible uniform and fine deposition stabilizing zinc anode at low temperature. Energy Storage Mater. **70**, 103489 (2024). <https://doi.org/10.1016/j.ensm.2024.103489>
3. N. Chang, T. Li, R. Li, S. Wang, Y. Yin et al., An aqueous hybrid electrolyte for low-temperature zinc-based energy storage devices. Energy Environ. Sci. **13**(10), 3527–3535 (2020). <https://doi.org/10.1039/d0ee01538e>
4. C. Xie, S. Liu, W. Zhang, H. Ji, S. Chu et al., Robust and wide temperature-range zinc metal batteries with unique electrolyte and substrate design. Angew. Chem. Int. Ed. **62**(28), e202304259 (2023). <https://doi.org/10.1002/anie.202304259>
5. P. Xiong, Y. Kang, N. Yao, X. Chen, H. Mao et al., Zn-ion transporting, *in situ* formed robust solid electrolyte interphase for stable zinc metal anodes over a wide temperature range. ACS Energy Lett. **8**(3), 1613–1625 (2023). <https://doi.org/10.1021/acsenergylett.3c00154>
6. S. You, Q. Deng, Z. Wang, Y. Chu, Y. Xu et al., Achieving highly stable Zn metal anodes at low temperature *via* regulating electrolyte solvation structure. Adv. Mater. **36**(26), 2402245 (2024). <https://doi.org/10.1002/adma.202402245>
7. D. Feng, F. Cao, L. Hou, T. Li, Y. Jiao et al., Immunizing aqueous Zn batteries against dendrite formation and side reactions at various temperatures *via* electrolyte additives. Small **17**(42), 2103195 (2021). <https://doi.org/10.1002/smll.202103195>
8. Z. Hou, Z. Lu, Q. Chen, B. Zhang, Realizing wide-temperature Zn metal anodes through concurrent interface stability regulation and solvation structure modulation. Energy Storage Mater. **42**, 517–525 (2021). <https://doi.org/10.1016/j.ensm.2021.08.011>
9. C. Lin, X. Yang, P. Xiong, H. Lin, L. He et al., High-rate, large capacity, and long life dendrite-free Zn metal anode enabled by trifunctional electrolyte additive with a wide temperature range. Adv. Sci. **9**(21), 2201433 (2022). <https://doi.org/10.1002/advs.202201433>
10. C. Song, Z. Gong, C. Bai, F. Cai, Z. Yuan et al., High performance Zn-I2 battery with acetonitrile electrolyte working at low temperature. Nano Res. **15**(4), 3170–3177 (2022). <https://doi.org/10.1007/s12274-021-3884-z>
11. J. Zhou, H. Yuan, J. Li, W. Wei, Y. Li et al., Highly reversible and stable Zn metal anode under wide temperature conditions enabled by modulating electrolyte chemistry. Chem. Eng. J. **442**, 136218 (2022). <https://doi.org/10.1016/j.cej.2022.136218>
12. J. Wang, Q. Zhu, F. Li, J. Chen, H. Yuan et al., Low-temperature and high-rate Zn metal batteries enabled by mitigating Zn^2+^ concentration polarization. Chem. Eng. J. **433**, 134589 (2022). <https://doi.org/10.1016/j.cej.2022.134589>
13. T. Wei, Y. Ren, Z. Li, X. Zhang, D. Ji et al., Bonding interaction regulation in hydrogel electrolyte enable dendrite-free aqueous zinc-ion batteries from −20 to 60 ℃. Chem. Eng. J. **434**, 134646 (2022). <https://doi.org/10.1016/j.cej.2022.134646>
14. X. Lu, Z. Liu, A. Amardeep, Z. Wu, L. Tao et al., Ultra-stable zinc metal anodes at-20 ℃ through eutectic solvation sheath in chlorine-functionalized eutectic electrolytes with 1, 3-dioxolane. Angew. Chem. Int. Ed. **62**(33), e202307475 (2023). <https://doi.org/10.1002/anie.202307475>
